# Scientometrics Approach to Research in Ovine Mastitis from 1970 to 2019 (with a Complete List of Relevant Literature References)

**DOI:** 10.3390/pathogens9070585

**Published:** 2020-07-17

**Authors:** Daphne T. Lianou, George C. Fthenakis

**Affiliations:** Veterinary Faculty, University of Thessaly, 43100 Karditsa, Greece; dlianou@vet.uth.gr

**Keywords:** ewe, intramammary infection, mastitis, meta-research, ovine, scientometrics, sheep, subclinical mastitis

## Abstract

The present study is a scientometrics evaluation of refereed publications on bacterial mastitis in sheep; the objectives were the evaluation of the relevant papers and the presentation of quantitative characteristics regarding their scientific content and bibliometric details. The Web of Science platform was used with search terms: [*mastitis* OR *mammary infection*] AND [*sheep* OR ewe* OR *ovine*] for papers from 1970 tο 2019; only ‘articles’, ‘reviews’, ‘proceedings papers’, or ‘data papers’ were evaluated, whilst documents related solely to contagious agalactia, mammary aspects of lentiviral infections, or infections of the teats and the udder skin were excluded. Finally, 580 papers were considered in detail. The number of published papers increased from 8 during the 1970s to 273 during the 2010s. These papers originated from 43 countries (most from Greece or Spain, n = 87 from each) and 240 institutions (145 universities and 95 other establishments), of which 35 produced ≥ 5 papers each. Most papers present original studies (n = 539) with a few reviews (n = 41). The original papers refer to dairy (n = 428), meat (n = 113), or wool (n = 1) production systems and present field (n = 329), laboratory (n = 163), or experimental (n = 67) work; the papers report aetiology (n = 146), risk factors (n = 100), pathogenesis (n = 92), diagnosis (n = 88), effects (n = 66), treatment (n = 50), control (n = 36), or descriptive epidemiology (n = 32) of the disease. Papers related to dairy production present more field and fewer experimental work than papers related to meat production; also, in papers describing work performed in dairy sheep, studies about aetiology, risk factors, and diagnosis of the disease predominate, whilst in papers performed in meat sheep, studies about aetiology, pathogenesis, and effects/diagnosis are reported more often. The papers were published in 175 scientific journals (most in *Small Ruminant Research*, n = 90, or *Journal of Dairy Science*, n = 54). On average, the papers received 16.8 total citations and 1.6 yearly citations (*h*-index = 47). Most papers were published in Scimago classification Q1 (n = 240) or Q2 (n = 230) journals and received 23.4 or 15.4 total citations, respectively. Reviews received more citations than original papers; among the latter, papers with work referring to dairy production received more yearly citations than papers referring to meat production; no differences in citations were seen according to type of work or mastitis aspect covered. Most citations were received by papers from France. Papers published in *Journal of Dairy Science* or *Small Ruminant Research* received the most citations. In total, there were 1558 individual authors of the papers, with 24 authors having co-authored > 10 papers each (max: 73 papers); on average, there were 5.2 co-authors per paper (min–max: 1–25). Average number of co-authors progressively increased from 2.1 in the 1970s to 6.3 in the 2010s, with original papers having a higher number of co-authors than reviews: 5.3 and 3.7, respectively. Papers from France had highers number of co-authors (7.9). The findings of this first ever scientometrics study into ovine mastitis indicate that the disease has not been studied as other sheep diseases and that future studies in it should be directed to its control.

## 1. Introduction

Scientometrics refers to the study of measuring and analysing scientific literature. Specific topics within this field refer to the measurement of the impact of research papers, the understanding of scientific citations, and the use of the results of such assessments in policy and management contexts [1]. There are overlaps between scientometrics and other scientific fields, e.g., meta-science. Meta-science can be defined as the use of research methods to study and appraise research itself and the areas where improvements can be made; meta-science studies can deal with methods, reporting, reproducibility, evaluation, and incentives, which are related with performance, communication, verification, evaluation, and rewarding research, respectively [2].

Scientometrics and meta-science papers produce new data by using information in previously published research articles. Such articles present features and characteristics of scientific research, with the aim to produce a quantitative assessment of the initial research papers. They differ from review papers, which summarise the state of knowledge on a topic, without reporting new facts or carrying out new analyses. During assessment of the relevant literature, authors of reviews may discuss research papers and provide expert opinions by combining findings and ideas from the primary sources.

In sheep, mammary infections can lead to bacterial mastitis (“mastitis”), mycoplasma mastitis (contagious agalactia), and lentiviral mammary infection. Among these, bacterial mastitis (“mastitis”) is the most important. It causes significant adverse financial consequences in sheep flocks and also reduces standards of welfare of sheep [3,4]. In recent years, various review papers on ovine mastitis have been published, presenting an overview or specific aspects of the disease. These reviews do not provide quantitative data of the relevant publications, which in turn could provide further insight into the disease. In fact, a scientometrics study of ovine mastitis has never been published.

The present study is a scientometrics evaluation of publications on bacterial mastitis (“mastitis”) in sheep during the last 50 years, from 1970 to 2019. Objectives of the study were the evaluation of the relevant publications and the presentation of quantitative characteristics regarding their scientific content and bibliometric details.

## 2. Materials and Methods

### 2.1. Search Procedure

The Web of Science platform (www.webofknowledge.com; Clarivate Analytics, Philadelphia, USA) was used for the search of relevant publications. Only publications in this platform were assessed and included in the study. Search terms employed were: [*mastitis* OR *mammary infection*] AND [*sheep* OR ewe* OR *ovine*]. Timespan was set to ‘*Custom year range (1970–2019)*’. The following Web of Science collections were included in the search: (i) Science citation index expanded 1970–2019 (1970 being the earliest year for which relevant information is provided by the platform) and (ii) Emerging sources citation index 2015–2019.

The initial search produced 1379 records. Document analysis of these was performed and the following types of documents were excluded: ‘meeting abstracts’, ‘notes’, ‘letters’, ‘editorial material’, ‘news items’, ‘corrections’, ‘early access papers’, and ‘reprints’. After their exclusion, a total of 1326 records remained, classified as ‘articles’ (n = 1250), ‘reviews’ (n = 76), ‘proceedings papers’ (n = 43), and ‘data papers’ (n = 1), for further assessment.

The abstracts of all these 1326 records were read and evaluated in the platform. Papers that did not deal with mastitis in sheep (e.g., papers not including sheep work, papers not including work on mammary diseases or their causal organisms) were excluded. Further, papers related solely to contagious agalactia or the mammary aspects of lentiviral infections were also excluded. Moreover, papers referring solely to infections of the teats and the udder skin (e.g., *Papilloma virus* infection of teats, contagious ecthyma, staphylococcal dermatitis, sheep pox) with no reference or association to bacterial mastitis were also excluded.

### 2.2. Paper Evaluation

After the above abstract review, 580 papers were considered for detailed evaluation. All these were individually assessed. In each paper, the following details were recorded.

- Year of publication.
- Journal in which it was published.
- Country and scientific establishment of origin (the country(ies) and the scientific establishment(s) of only the first and the last authors were taken into account).
- Number of literature references included in the relevant list.
- Total number of citations received by the paper until the end of 2019.
- Number and names of all co-authors.
- Type of paper: (i) original (paper presenting and providing new information, e.g., results or analyses, including case reports) or (ii) review (paper surveying and summarizing previously published studies, with no presentation and report of new facts or analyses). For original papers, the following details were also recorded.
  ▪ Sheep production system to which the work referred: (i) dairy, (ii) meat, or (iii) wool; all systems that applied were assigned per paper.
  ▪ Type of study described in the paper: (i) experimental (study performed predominantly with animals and involving experimental work, e.g., bacterial inoculations, allocation of animals to groups), (ii) field (study performed predominantly with animals and not involving experimental work, e.g., clinical work, survey work, farm work), or (iii) laboratory (study performed predominantly without animals, although material on which it was based would have originated from animals); only one of these three types was assigned per paper.
  ▪ Mastitis aspect described in the paper: (i) aetiology, (ii) control, (iii) descriptive epidemiology, (iv) diagnosis, (v) effects, (vi) pathogenesis, (vii) risk factors, or (viii) treatment; up to two of these were assigned per paper.

### 2.3. Data Management and Analysis

All data were entered into Microsoft Excel. Descriptive analysis was performed initially. The frequency of the various outcomes was evaluated in tables of cross-categorised frequency data by use of the Pearson chi-square test as appropriate. Comparisons between continuous data were performed by use of one-way analysis of variance. Correlations were performed as indicated and significance of the result was evaluated. Statistical significance was defined at *p* < 0.05.

## 3. Results

All the 580 papers individually assessed are indexed in the Web of Science, fulfil the search criteria, and present work on ovine mastitis. A complete list of these papers with their details is in Table S1.

### 3.1. Year of Publication and Origin of Papers

**Figure 1:**
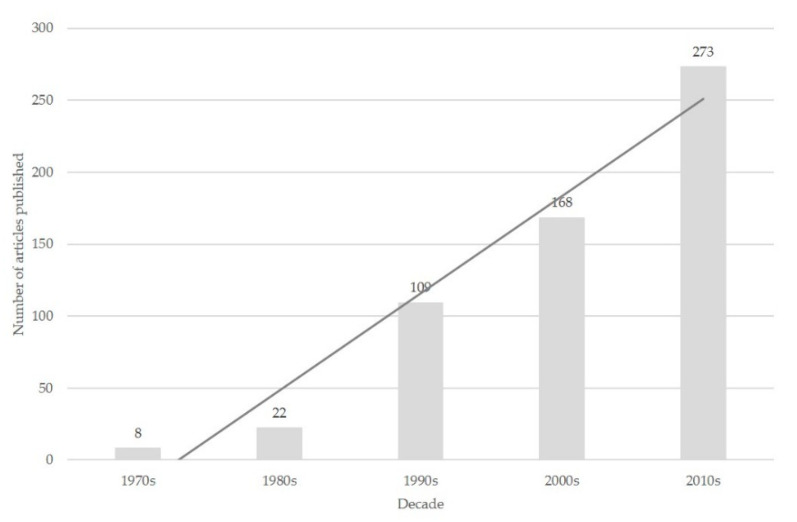
Number of papers published on mastitis in sheep from 1970 to 2019, classified according to the decade during which they were published.

The number of papers published per decade in the topic increased progressively: 8 (1.4%) papers were published in the 1970s, 22 (3.8%) in the 1980s, 109 (18.8%) in the 1990s, 168 (28.9%) in the 2000s, and 273 (47.1%) in the 2010s (Figure 1).

Papers originated from 43 different countries (Table S2). Most papers originated from Greece or Spain (n = 87 from each, 15.0%), Italy (n = 78, 13.4%), United Kingdom (n = 50, 8.6%), Brazil (n = 40, 6.9%), France (n = 31, 5.3%), United States of America (n = 26, 4.5%), Australia (n = 24, 4.1%), Israel (n = 23, 4.0%), and Austria or Turkey (n = 17 from each, n = 2.9%). Three hundred two papers originated from the first four countries above (52.1% of all papers), while 480 papers were from the above 11 countries (82.8%). When geographical regions were considered, it was found that most papers originated from the countries of Southern Europe (n = 303, 52.2% of all papers), the countries of Central and Northern Europe (n = 85, 14.7%), and the countries of the Middle East (n = 63, 10.9%).

**Figure 2:**
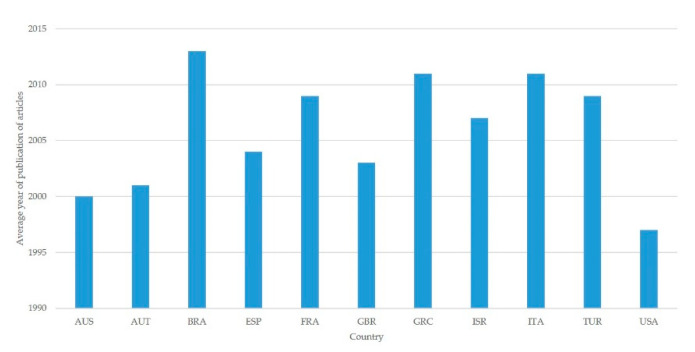
Average year of publication of papers on mastitis in sheep from 1970 to 2019 from the 11 countries with the most papers published (abbreviations of country names according to International Naming Convention ISO 3166).

Differences in the year of publication are significant between the above 11 countries (*p* < 0.001). Papers from Brazil, Greece, and Italy are the most recent ones (average year of publication: 2013, 2011, and 2011, respectively), whilst papers from Australia and the United States of America are the oldest ones (average year of publication: 2000 and 1997, respectively) (Figure 2).

Papers originated from 240 different scientific establishments around the world, of which 35 published at least 5 papers each (Table S3). Of these 35 establishments, 31 (88.6%) are located in the 11 countries with highest numbers of published papers. Among all the establishments, 145 are universities (26 of these produced ≥ 5 papers), from which originated 540 papers (93.1%); the other 95 include research institutes, services of Ministries of Agriculture, pharmaceutical laboratories, veterinary practices, etc. (9 of these produced ≥ 5 papers), from which originated 236 papers (40.7%) (*p* < 0.0001). The establishments that produced more papers are the University of Thessaly (n = 64; 11.0%), the University of Leon, the University of London (n = 25 each; 4.3%), the Aristotle University of Thessaloniki, the National Institute of Agronomic Research France (n = 21 each; 3.6%), the Complutense University of Madrid, the Veterinary University of Vienna (n = 18 each; 3.1%), the Kimron Veterinary Institute Israel (n = 17; 2.9%), the Commonwealth Scientific and Industrial Research Organisation Australia (n = 16; 2.8%), and the University of Palermo (n = 15; 2.6%).

### 3.2. Type and Content of Papers

**Figure 3:**
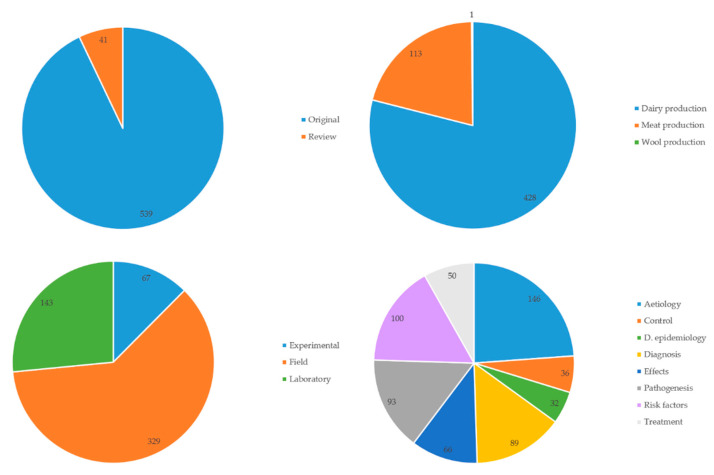
Frequency of published papers on mastitis in sheep from 1970 to 2019 according to type and content (**top left**: types of papers; **top right**: types of production system to which the original papers referred; **bottom left**: types of studies in original papers; **bottom right**: mastitis aspects reported in the original papers—colour legend for each pie diagram to its right).

**Figure 4:**
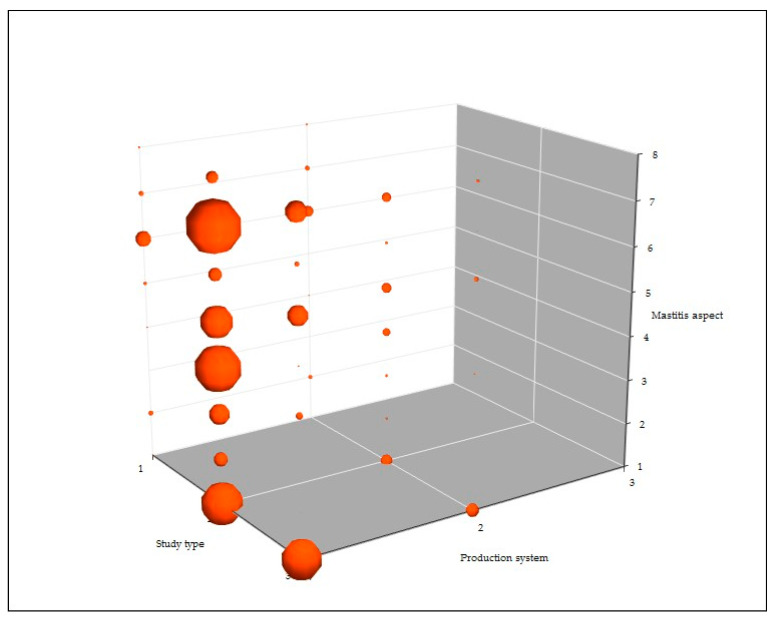
Association between type of study, production system referred to, and mastitis aspect in papers on ovine mastitis published during the period 1970–2019 (Study type: 1 experimental, 2 field, 3 laboratory; Production system: 1 dairy, 2 meat, 3 wool; Mastitis aspect: 1 aetiology, 2 control, 3 epidemiology, 4 diagnosis, 5 effects, 6 pathogenesis, 7 risk factors, 8 treatment. Size of the spheres reflects number of papers at each point).

Most papers presented original studies (n = 539; 92.9%), and fewer ones presented reviews (n = 41; 6.1%). Most original papers referred to dairy production (n = 428; 79.4%), with fewer ones referring to meat (n = 113; 21.0%) or wool (n = 1) production. Most original papers presented field work (n = 329; 61.0%), and fewer ones presented laboratory (n = 143; 26.5%) or experimental (n = 67; 12.4%) work. Original studies reported mostly aetiology (n = 146; 27.1%), risk factors (n = 100; 18.6%), pathogenesis (n = 93; 17.3%), or diagnosis (n = 89; 16.8%) of the disease, whilst fewer papers existed on descriptive epidemiology (n = 32; 5.9%), control (n = 36; 6.7%), treatment (n = 50; 9.3%), or effects (n = 66; 12.2%) of the disease (Figure 3 and Figure 4, Table S4).

There are clear differences in the type of work presented in papers according to the production system to which the study referred. Papers related to dairy production presented proportionately more field and fewer experimental work than papers related to meat production; for dairy production, the number of papers presenting field, laboratory, and experimental work were 272, 113, and 43, respectively (63.6%, 26.4%, and 10.0%), whilst respective figures for papers related to meat production sheep were 58, 31, and 24 (51.3%, 27.4%, and 21.2%) (*p* = 0.0036).

There were also some differences in the mastitis aspect reported in the papers according to the production system. In papers performed in dairy sheep, studies about aetiology, risk factors, and diagnosis predominated (n = 111, 86, and 74, respectively; 25.9%, 20.1%, 17.3%), whilst in papers performed in meat sheep studies about aetiology, pathogenesis, and effects/diagnosis were reported more often (n = 35, 26, and 15, respectively; 31.0%, 23.0%, 13.3%) of the disease (*p* = 0.044).

There were also clear differences between the 11 countries with the most papers, in the type of paper published (*p* = 0.0065), in the production system being referenced (*p* < 0.0001), in the type of studies performed (*p* < 0.0001), and in the mastitis aspect presented (*p* < 0.0001). Similar differences were also recorded when only the 6, the 4, or the 3 countries with the most papers were included in the analysis (*p* < 0.008 in all cases) (Table S5).

### 3.3. Journals

In total, the 580 papers were published in 175 scientific journals. Journals in which over 10 papers were published are the following: *Small Ruminant Research* (n = 90, 15.5%), *Journal of Dairy Science* (n = 54, 9.3%), *Veterinary Microbiology* (n = 29, 5.0%), *Journal of Dairy Research* (n = 22, 3.8%), *Pesquisa Veterinária Brasileira* and *The Veterinary Record* (n = 14 each, 2.4%), *The Veterinary Journal* (including *British Veterinary Journal*), and *Journal of Veterinary Medicine B* (including *Zentralblatt für Veterinärmedizin Reihe B*) (n = 11 each, 1.9%); cumulatively, 245 papers (42.2%) were published in these 8 journals.

**Figure 5:**
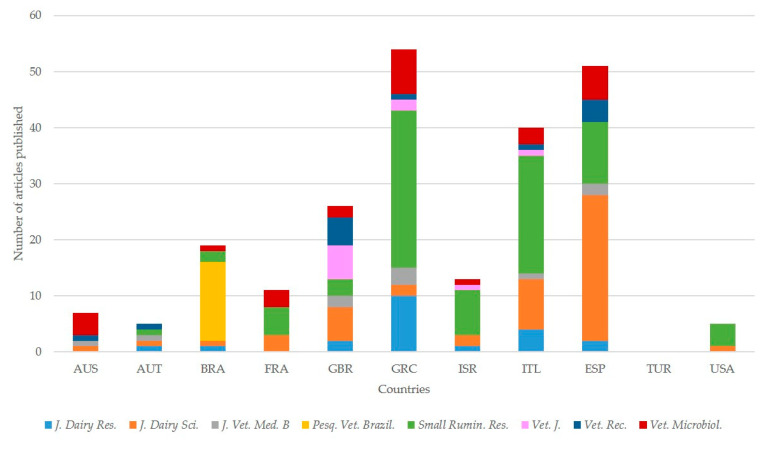
Association between journals and countries of origin of papers on ovine mastitis published during the period 1970–2019 (abbreviations of country names according to International Naming Convention ISO 3166; abbreviations of journals from left to right: *Journal of Dairy Research*, *Journal of Dairy Science*, *Journal of Veterinary Medicine B, Pesquisa Veterinária Brasileira*, *Small Ruminant Research*, *The Veterinary Journal*, *The Veterinary Record*, *Veterinary Microbiology*).

In total, 225 of the 245 papers in these journals (91.8%) originated from 10 of the 11 countries with the most papers (i.e., except Turkey); in contrast, if all articles are taken into account, a smaller proportion (463 of 580; 79.8%) originated from these 10 countries (*p* < 0.0001s). There was no further pattern of statistical association between these 8 journals and the 11 countries with the most publications (*p* > 0.95) (Figure 5, Table S6).

**Figure 6:**
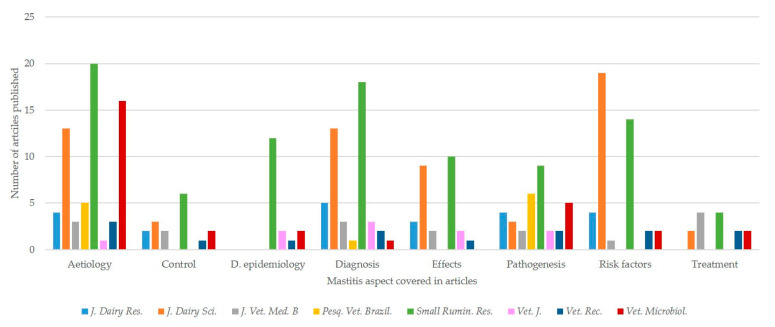
Association between journals and aspect of mastitis covered in papers on ovine mastitis published during the period 1970–2019 (abbreviations of journals from left to right: *Journal of Dairy Research*, *Journal of Dairy Science*, *Journal of Veterinary Medicine B, Pesquisa Veterinária Brasileira*, *Small Ruminant Research*, *The Veterinary Journal*, *The Veterinary Record*, *Veterinary Microbiology*).

When the type and content of papers were considered between these eight journals, there were significant differences in the type of papers published (*p* = 0.045) as well as in the production system being referenced, the type of studies performed, and the mastitis aspect presented (*p* < 0.001 for all comparisons). Similar significant differences were recorded also when the comparisons were made with the findings of only the 4 or the 3 journals with the most papers included in the analysis (Figure 6, Table S7).

On average, the papers include 33.3 ± 1.0 literature references (mean ± standard error of the mean). Papers published since 2000 included significantly more references than those published earlier (*p* < 0.0001). Review papers included significantly more references than did original papers, 81.6 versus 29.6, respectively (*p* < 0.0001); moreover, papers describing laboratory work included more references than ones describing experimental work and field work: 32.2, 30.7, and 28.2, respectively (*p* = 0.0310), but no differences existed between papers according to mastitis aspect therein (*p* = 0.48) (Table S8).

### 3.4. Impact of Papers

The papers received (until the end of 2019) on average 16.8 ± 1.0 total citations and 1.6 ± 0.1 citations per year in the Web of Science platform. For all the papers, *h*-index was 47 and *i_10_*-index was 280. There was a clear correlation between the number of literature references in a paper and the number of citations received (*r* = 0.224 for correlation with the total number of citations, *r* = 0.456 for correlation with the number of citations per year, *p* < 0.0001). When original and review papers were considered separately, a correlation was also evident between references and yearly citations (*r* = 0.400 for original papers and *r* = 0.369 for reviews, *p* < 0.011), but not between references and total citations (*r* = 0.058 and *r* = 0.242, respectively, *p* > 0.055).

With regard to Scimago classification (year: 2019), most papers were published in Q1 (n = 240) or Q2 (n = 230) journals, with fewer ones in Q3 (n = 63) or Q4 (n = 22) journals or in journals outside this classification (n = 25). Papers in Q1 journals received more citations and more yearly citations than papers in Q2 or in Q3/Q4 or outside the classification journals: 23.4 ± 2.0 and 2.3 ± 0.2, 15.4 ± 1.5 and 1.4 ± 0.1, and 5.3 ± 0.5 and 0.5 ± 0.1, respectively (*p* < 0.0001).

More citations were received by papers published in the last 20 years (*p* < 0.0002). Most citations (total citations per paper, total yearly citations per paper, total cites) were received by papers with an origin in France; these were published mostly (74.0%) in Q1 journals (*p* < 0.0005). Also, papers with origins from establishments in France (National Institute of Agronomic Research, University of Toulouse) were the ones with most total citations and total yearly citations; these establishments also published papers mostly in Q1 journals (≥80%). However, establishments outside France (University of Leon and University of Thessaly) were the ones with most total cites (Table S9).

**Figure 7:**
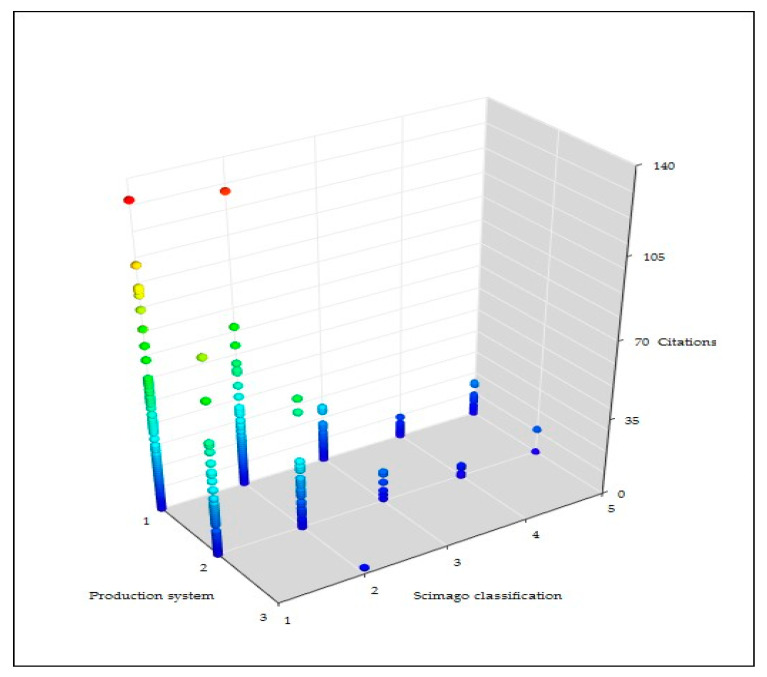
Association between production system referred to in papers, journal classification, and citations in papers on ovine mastitis published during the period 1970–2019 (Production system: 1 dairy, 2 meat, 3 wool; Scimago classification: 1 Q1, 2 Q2, 3 Q3, 4 Q4, 5 not included in the database).

On average, reviews received more total citations and total yearly citations per paper (35.4 ± 9.9 and 3.6 ± 0.7, respectively) than did original papers (15.4 ± 0.8 and 1.4 ± 0.1) (*p* < 0.0001), but fewer total cites (1450 and 8296, respectively). Papers with work referring to dairy production received significantly more yearly citations than did papers referring to meat production, although no differences were evident in the distribution according to journal classification (Figure 7). With regard to type of study, although significantly more experimental or laboratory (55.2% and 51.7%, respectively) than field (35.3%) works were published in Q1 journals (*p* = 0.0006), no significant differences in the citations received between papers of the three types were found (*p* > 0.59). With regard to mastitis aspect, differences were not significant in the classification of the journals in which papers were published (*p* = 0.067); also, differences in the average yearly citations received by papers did not differ according to the mastitis aspect (*p* = 0.407). Details are in Table S9.

Papers published in *Journal of Dairy Science* or *Small Ruminant Research* received the most citations (*p* < 0.02 for comparisons between journals). There were no correlations between the Scimago classifications of the journals and the citations received by papers published therein (*p* > 0.13 for all comparisons) (Table S10).

### 3.5. Authors of Papers

In total, there were 1559 individual authors of the papers; among these, 24 authors co-authored over 10 papers each (max: 73). Cumulatively, in the 580 papers, there were 2993 co-authors, i.e., on average 5.2 ± 0.1 co-authors per paper (median: 5, min-max: 1–25). Furthermore, there were 640 individual authors who were first or last authors in the papers; among these, 6 authors were first or last in over 10 papers each (max: 62). On average, the 24 authors with >10 papers each were first or last authors in 53.0 ± 6.1% of the papers in which they were co-authors (min: 0.0%, max: 90.9%).

The average number of co-authors per paper progressively increased from 2.1 ± 0.4 and 2.6 ± 0.2 in the 1970s and 1980s, respectively, to 3.7 ± 0.2 and 4.8 ± 0.2 in the 1990s and 2000s, respectively, to 6.3 ± 0.2 in the 2010s (*p* < 0.0001). In 30 papers (5.2%), there was only one author.

Moreover, there was a clear difference in the number of authors in the papers from the various countries (*p* < 0.0001); Papers from France, Italy, and Greece had the highest number of co-authors (7.9 ± 0.9, 6.4 ± 0.4, and 6.1 ± 0.1, respectively), whilst papers from Australia and the United Kingdom had the lowest (3.3 ± 0.3 and 3.2 ± 0.3). All the above 24 authors with the most papers worked in one of the 11 countries (most in Greece, n = 10) and in one of the 35 establishments (most in University of Thessaly, n = 8) from which most papers originated. Among them, 2 authors published papers with origins from two different countries and 4 authors published papers from two different establishments.

Original papers had a significantly higher number of co-authors than did reviews: 5.3 ± 0.1 versus 3.7 ± 0.3 (*p* = 0.0008). In contrast, there was no difference in the number of co-authors among the papers according to study type (*p* = 0.15) or mastitis aspect (*p* = 0.54) therein.

**Figure 8:**
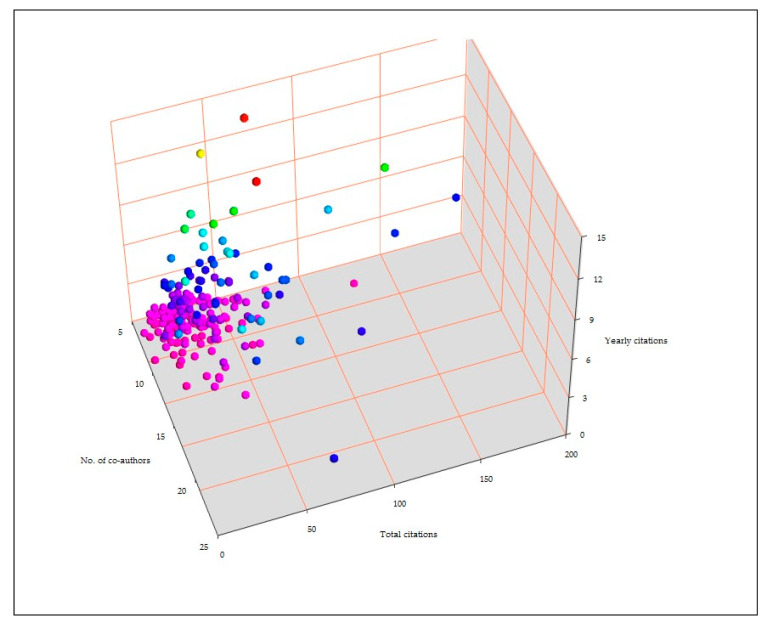
Association between number of co-authors in papers with total citations and yearly citations received by these papers.

There was a clear correlation between number of co-authors and yearly citations per paper (*r* = 0.228, *p* < 0.0001) but not with total citations per paper (*r* = −0.025, *p* = 0.27). When only papers with > 5.2 authors (i.e., the average co-authors per paper) were taken into account, correlation was seen for both parameters (*r* = 0.226, *p* = 0.0003 and *r* = 0.208, *p* = 0.0009, respectively) (Figure 8). Papers with one of the above 24 authors as first or last author received significantly more citations than other papers: 20.6 ± 2.0 total citations and 2.0 ± 0.2 yearly citations versus 15.1 ± 1.2 and 1.5 ± 0.1, respectively (*p* = 0.0098 and 0.0033, respectively).

## 4. Discussion

Ovine mastitis is an important disease of sheep. The disease leads to significantly reduced production in affected animals and adversely affects their welfare standards.

Probably the earliest reference to ovine mastitis was in a French language dictionary by the French veterinarian Hurtrel d’ Arboval in 1823 [5] in the lemma “araignée” (spider). He described an acute fatal inflammation of the mammary gland of ewes and mentioned that the disease was erroneously believed to be due to the bite of an “insect“. He stated that the disease was more often present in unsheared than in sheared ewes. He mentioned that the disease was called among French shepherds “mal de pis” (evil of the udder) or “l’ araignée” (the spider). Nevertheless, the first known scientific approach to investigating ovine mastitis was undertaken by the French veterinarian and microbiologist Edmund Nocard in 1886. Nocard [5] quoted the experience of a practicing veterinarian about the disease, described in detail the sequence of signs of the disease, and performed detailed pathological and microbiological examinations. He isolated from the milk of an affected ewe a micrococcus and described its morphological, cultural, and biochemical characteristics. He described how experimental intramammary inoculation of secretion of diseased mammary gland or of a broth culture via the teat canal always successfully reproduced a condition very similar to the natural disease. Bridré [6] undertook a field investigation among hand-milked ewes and found that the incidence of the disease was approximately 5% and the case fatality 20%. He was the first who attempted to protect sheep against experimentally induced mastitis by means of immunization. Leyshon [7] was the first to investigate the disease in lactating ewes in England, in the region of East Anglia; he isolated from the mammary secretion of the diseased ewes a microorganism, whose morphological characteristics were different from the micrococcus isolated by Nocard (later identified as *Mannheimia haemolytica*). Minett [8] gave an account of the first reports on ovine mastitis and reviewed references on the occurrence and the aetiology of the disease. Later, Pegreffi [9] gave an account of the subsequent literature and Landau and Tamarin [10] reviewed the literature of the period 1963–1974.

Despite its importance, mastitis in sheep has not been adequately studied, and there are facets of the disease that still have not been clarified. There are no scientometrics studies available in the international literature that would allow comparison of the mastitis literature to that about other diseases of sheep. An initial stage search revealed 2099 records (‘articles’, ‘reviews’, ‘proceedings papers’, and ‘data papers’) for [respiratory infection* OR *pneumonia*] and 1670 records for [*abortion*] during the same year range as in the current study (for comparison: 1326 records found in the present study at the respective stage of the search). Also, there are no relevant studies to compare the present findings to those for mastitis in other species. Again, an initial stage search as above revealed 11,196 records for [*cattle* OR cow* OR *bovine*] and 1504 documents for [goat* OR doe* OR *caprine*]. These findings lend weight to the idea that mastitis in ewes has not been studied extensively.

Nevertheless, the progressive increase of published papers indicates that this lack of scientific work has been identified by researchers, and relevant studies have been designed and performed. Possibly, the recognition by the European Food Safety Authority [3] of the significance of mastitis as a very important disease of sheep might have contributed to the increase of relevant publications in recent years.

Most published papers originated from countries in the para-Mediterranean area. This was expected given that a significant number of sheep exist in these countries (approximately 15% of the world’s sheep population) [11]. Sheep production systems in these countries are directed towards milk production, which also explains the increased number of papers referring to dairy production and indicates research oriented towards specific needs of agriculture in these countries. Consequently, most papers also originated from scientific establishments in these countries.

As sheep production in the para-Mediterranean area is oriented towards dairy production, one may indicate that the adverse effects of mastitis (e.g., reduced milk production, changes in milk quality) [4] become evident immediately. In contrast, possible effects in flocks with meat production as the primary objective (e.g., reduced growth rate of lambs) [12] cannot be seen as easily, which undermines the recognition of the importance of the disease and hence the development of relevant studies.

The findings indicate that some aspects of the disease have been covered extensively, e.g., aetiology, risk factors, and diagnosis. This is reflected in the presence in the international literature of papers that reviewed specific facets of the disease, e.g., [13,14,15], or methodologies employed for its study, e.g., [16]. However, other aspects of the disease, especially its control, have not been studied adequately, as evidenced by the small number of papers covering these topics. Hence, findings of scientometrics studies are useful and can be taken into account by governments, grant-giving bodies, and researchers to direct resources of research and perform relevant studies accordingly. Control studies into ovine mastitis can thus be a research priority for the near future.

The findings also indicate the accumulation of research on ovine mastitis in some establishments. This is a trend that is observed with many research topics. Hosting institutions and researchers accumulate expertise on a topic and capitalize upon it to further expand their work. The presence of large groups also seems to favour scientific output; among the authors with many papers on the subject, most originated from the same establishment, which indicates not only large groups but also long-established ones play a role in supporting the increased number of publications from those groups and of citations to those publications.

The journals in which the papers were mostly published are ones with a specific approach to sheep (*Small Ruminant Research*) or a thematic approach to dairy studies (*Journal of Dairy Research*, *Journal of Dairy Science*) rather than journals of general interest. This indicates the increased importance of topical scientific journals and the preference of researchers to publish in them; in that way, researchers address themselves to a more specialised audience with greater interest in their work and the ability to understand it.

In conclusion, this is the first ever scientometrics evaluation of mastitis in sheep. The findings indicate that the disease has not been studied as much as other diseases of sheep. Future studies should be directed to the control of the disease by using knowledge obtained from work into its other facets.

## Supplementary Materials

The following are available online at https://www.mdpi.com/2076-0817/9/7/585/s1, Table S1: Details of 580 papers published from 1970 until 2019 on ovine mastitis and listed in the Web of Science platform; Table S2: Alphabetical list of countries from which papers on ovine mastitis have been published during the period –2019; Table S3: Alphabetical list of scientific establishments from which at least 5 papers on ovine mastitis have been published during the period 1970–2019; Table S4: Classification of papers on ovine mastitis published during the period 1970–2019, according to paper type, sheep production system, study type and mastitis aspect covered therein. Table S5: Number of papers on ovine mastitis published during the period 1970–2019 originating from the 11 countries with highest number of papers published, classified according to paper type, sheep production system, study type and mastitis aspect covered. Table S6: Associations between journals and countries of origin of papers on ovine mastitis published during the period 1970–2019; Table S7: Number of papers on ovine mastitis published during the period 1970–2019 in the 8 journals with the majority of relevant papers, classified according to paper type, sheep production system, study type and mastitis aspect covered. Table S8: Literature references in papers on ovine mastitis published during the period 1970–2019, according to year of publication, country of origin, establishment of origin, journal in which published and characteristic of paper. Table S9: Citations received by papers on ovine mastitis published during the period 1970–2019, and impact of the respective journal, according to year of publication, country of origin, establishment of origin, journal in which published and type of paper, production system, type of study and aspect of mastitis covered. Table S10: Citations received by papers on ovine mastitis published during the period 1970–2019, according to the journal in which they have been published.
