# Supplementary material for: Scientometrics Approach to Research in Ovine Mastitis from 1970 to 2019 (with a Complete List of Relevant Literature References)
Source: Pathogens. 2020 Jul 17;9(7):585. doi: 10.3390/pathogens9070585 (PMC7399971; doi:10.3390/pathogens9070585)
Supplement: Supplementary file 1 [file pathogens-09-00585-s001.pdf]

Communication

Scientometrics Approach to Research in Ovine Mastitis from 1970 to 2019 (with a Complete List of Relevant Literature References)

Daphne T. Lianou and George C. Fthenakis

Table S1. Details of 580 papers published from 1970 until 2019 on ovine mastitis and listed in the Web of Science platform

No.	Title of article	Year of publication	Journal in which published	First author	No. of authors	Last author	Country(ies) of origin		Establishment(s) of origin		Article type	Study type	Mastitis aspect covered		Production type referred to
1	Experimental ovine mastitis -A pathologic study	1970	Vet Pathol	El-Etreby	2	Abdel-Hamid	EGY		U Assiut		Original	Experimental	Pathogenesis		Dairy
2	Experimental production of mastitis in sheep by Mycobacterium smegmatis and Mycobacterium fortuitum	1971	Cornell Vet	Richardson	1		USA		U Cornell		Original	Experimental	Pathogenesis		Meat
3	Mastitis produced experimentally in sheep with ovine abortion Chlamydia	1972	Zblatt Vetmed B	Papadopoulos	2	Leontides	GRE		Aristotle U Thessaloniki		Original	Experimental	Pathogenesis		Dairy
4	Bacillus cereus as a cause of bovine mastitis 1. Occurrence of a case of mastitis and studies on its infectivity for sheep and cow	1973	Zblatt Vetmed B	Merck	2	Burrow	FRG		U Berlin		Original	Experimental	Pathogenesis		Meat
5	Observation of a spontaneous case of fatal ovine mastitis due to Klebsiella pneumoniae	1977	Zblatt Vetmed B	Mandal	3	Ahuja	IND		U Punjab		Original	Field	Aetiology		Dairy
6	Mastitis in cows, goats and sheep	1978	Norsk Vetmed	Mosdol	1		NOR		U Oslo		Original	Field	Aetiology		Meat
7	Incidence and importance of subclinical mastitis in sheep	1978	J Anim Sci	Gross	4	Torell	USA		U California Davis		Original	Field	Aetiology		Meat
8	Genetic and environmental effects on milk production, milk composition and mastitis incidence in crossbred ewes	1979	J Anim Sci	Torres-Hernandez	2	Hohenboken	USA		U Oregon		Original	Field	Risk factors		Dairy
9	Mastitis and other abnormalities as related to neonatal lamb mortality in shed-lambed range ewes	1980	J Anim Sci	Kirk	3	Anderson	USA		U Idaho		Original	Field	Effects		Meat
10	Prevantion of post weaning mastitis in ewes	1981	Vet Rec	Hendy	4	Davies	GBR		Glaxo		Original	Field	Control		Meat
11	Immunization against experimental staph. Mastitis -Effect of challenge with a heterologous strain of Staphylococcus aureus	1981	Aus Vet J	Watson	2	Kennedy	AUS		U James Cook	CSIRO	Original	Experimental	Control		Meat
12	Effect of immunization of early influx of neutrophils during staphylococcal mastitis in sheep	1982	Res Vet Sci	Colditz	2	Watson	AUS		U Queensland	CSIRO	Original	Experimental	Control		Meat
13	The role of humoral and cellular mediators in enhanced mammary inflammatory reactions to staphylococcal infection in systemically immunised ewes	1982	Microbiol Immunol	Colditz	2	Watson	AUS		U Queensland	CSIRO	Original	Experimental	Control		Meat
14	Mastitis in sheep in the Netherlands	1983	Tijd DiergenK	Konig	3	Gelling	NED		Vakgroup Bedrijfsdiergeneeskunde & Buitenprakt		Original	Field	Aetiology		Meat
15	Ovine mastitis due to Histophilus ovis	1983	Can Vet J	Beaurgard	2	Higgins	CDN		U Montreal		Original	Field	Aetiology		Meat
16	Modern aspects of sheep mastitis	1984	Br Vet J	Watson	2	Buswell	GBR		Beecham Animal Health		Review				
17	Mastitis in sheep in Iraq	1985	Vet Rec	Al-Samarrae	3	Yousif	IRQ	IND	U Bagdhad	U Haryana	Original	Field	Aetiology		Dairy
18	Immunophysiological activity of supramammary lymph nodes of the ewe	1985	Res Vet Sci	Watson	2	Davies	AUS		U New England	CSIRO	Original	Experimental	Pathogenesis		Meat
19	Pathology of acute experimental of Actinobacillus seminis mastitis	1985	Aus Vet J	Alsenosy	2	Dennis	USA		U Cansas		Original	Experimental	Pathogenesis		Meat
20	Mastitis and cell content milk in milk from Scottish blackface ewes	1986	Vet Rec	Mackie	2	Rodgers	GBR		Veterinary Research Laboratory Northern Ireland		Original	Field	Diagnosis		Meat
21	Patterns of non-clinical intramammary infection in a ewe flock	1986	J Am Vet Med Assoc	Hueston	3	Judy	USA		U Ohio State		Original	Field	Aetiology		Meat
22	Detection of ovine intramammary infection	1986	J Am Vet Med Assoc	Hueston	3	Judy	USA		U Ohio State		Original	Field	Diagnosis		Meat
23	Diseases of sheep and goats: mastitis	1987	Wiener Tierarzt Monats	Sasshofer	3	Kessler	AUT		U Vienna		Review				
24	Iatrogenic mastitis in sheep	1987	Tijd DiergenK	Roumen	3	Gruys	NED		Gezondheidsienst Dieren		Original	Field	Pathogenesis		Meat
25	Detection of subclinical mastitis in ewes	1987	Br Vet J	Maisi	3	Seppanen	FIN		U Helsinki		Original	Field	Diagnosis		Meat
26	Protective effect of glucan against experimentally induced staphylococcal mastitis in ewes	1988	Vet Microbiol	Buddle	3	Ralston	NZL		Ministry of Agriculture New Zealand		Original	Experimental	Control		Meat
27	Vaccination against experimental mastitis	1988	Res Vet Sci	Watson	1		AUS		CSIRO		Original	Experimental	Control		Meat
28	Incidence and control of subclinical mastitis in intensively managed ewes	1988	J Anim Sci	McCarthy	4	Notter	USA		U Virginia Polytechnic		Original	Field	Epidemiology		Meat
29	Non clinical intramammary infection in lactating ewes and its association with clinical mastitis	1989	Br Vet J	Bor	3	Gootwine	ISR		Agri Research Organisation Israel		Original	Field	Epidemiology		Dairy
30	Intramammary antibiotic treatment at the end of lactation for prophylaxis and treatment of intramammary infections in ewes	1989	J Am Vet Med Assoc	Hueston	3	Baertsche	USA		U Ohio State		Original	Field	Control		Meat
31	Investigations on cell counts of milk and distribution of bacterial mastitis in lower Austrian sheep and goat flocks	1990	Wiener Tierarzt Monats	Deutz	4	Baumgartner	AUT		U Vienna		Original	Field	Epidemiology		Dairy
32	Incidence of staphylococci in ovine mastitic milk and antibiotic susceptibility of the strains	1990	MilchWissenSchaft	Gutierrez	5	Moreno	FRA		U Leon		Original	Field	Epidemiology	Treatment	Dairy
33	The effect of experimentally induced of subclinical mastitis on milk yield of ewes and on the growth of lambs	1990	Br Vet J	Fthenakis	2	Jones	GBR		U London		Original	Experimental	Effects		Meat
34	Survey of intramammary infections in ewes on the New England Tableland of New South Wales	1990	Aus Vet J	Watson	5	Frost	AUS		U Queensland	CSIRO	Original	Field	Epidemiology		Meat
35	Acute mastitis in sheep	1990	Prakt Tierarzt	Bostedt	1		FRG		U Giessen		Original	Field	Epidemiology		Meat
36	Milk whey induction of agglutination in ovine and bovine mastitis Staphylooccus aureus	1990	Zblatt Vetmed B	Baselga	2	Amorena	ESP		U Zaragoza		Original	Laboratory	Aetiology		Dairy
37	The influence of different factors on milk composition and production of the ewes of the Tsigai breed within the milking period	1991	Zivocisna Vyroba	Margetin	4	Capistrak	CZE		Research Institute Sheep Breeding Czechia		Original	Field	Effects		Dairy
38	Somatic cells counts and CMT results in milk sheep with normal udder health during a complete lactation period	1991	Schweizer Arch Tierarzt	Regi	5	Rusch	SWI		U Zurich	Swiss Federal Institute of Technology	Original	Field	Diagnosis		Dairy
39	Suitability of lactate dehydrogenase activity and somatic cell counts of milk for detection of subclinical mastitis in Merino ewes	1991	Acta Vet Hung	Nizamioglou	2	Erganis	TUR		U Selcuk		Original	Laboratory	Diagnosis		Dairy
40	Infection of rabbit mammary glands with ovine mastitis bacterial strains	1991	J Comp Pathol	Amorena	10	Faris	ESP	SWE	SIA DGA-Zaragoza	U Uppsala	Original	Laboratory	Pathogenesis		Dairy
41	Variation in somatic cell counts, California Mastitis Test and electrical conductivity among various fractions of ewes milk	1991	J Dairy Sci	Peris	5	Torres	ESP		U Valencia		Original	Laboratory	Diagnosis		Dairy
42	A field investigation of subclinical mastitis in sheep	1991	Br Vet J	Watkins	3	Jones	GBR		U London		Original	Field	Epidemiology		Meat
43	The experimental production of mastitis in sheep by intramammary inoculation of Pasteurella haemolytica	1991	J Comp Pathol	El-Masannat	3	Scott	GBR		U London		Original	Experimental	Pathogenesis		Meat
44	Somatic cell counts of ewes milk	1991	Br Vet J	Fthenakis	4	Jones	GBR		U London		Original	Laboratory	Diagnosis		Meat
45	An efficient microtest to study adherence of bacteria to mammalian cells	1992	FEMS Microbiol Lett	Aguilar	4	Amorena	ESP		SIA DGA-Zaragoza		Original	Laboratory	Pathogenesis		Dairy
46	Enterotoxin and toxic shock syndrome toxin-one production by staphylococci isolated from mastitis in sheep	1992	APMIS	Orden	6	Delafuente	ESP		U Madrid		Original	Laboratory	Aetiology		Dairy
47	Production of staphylococcal enterotoxin and TSST-1 by coagulase-negative staphylococci isolated from ruminant mastitis	1992	Zblatt Vetmed B	Orden	6	Gomez-Lucia	ESP		U Madrid		Original	Laboratory	Aetiology		Dairy
48	Influence of the lactation period on the cell count of milk samples of ewes	1992	Deut Tierarzt Wochen	Baumgartner	3	Eibl	AUS		U Vienna		Original	Field	Diagnosis		Dairy
49	Nucleotide sequences and biologic properties of toxic shock syndrome from ovine-associated and bovine-associated Staphylococcus aureus	1992	J Infect Dis	Lee	9	Schlievert	USA		U Minnesota		Original	Laboratory	Aetiology		Meat
50	Detection of enterotoxin and TSST-1 secreted by Staphylococcus aureus isolated from ruminant mastitis -Comparison of ELISA and immunoblot	1992	J Appl Bacteriol	Orden	6	Gomez-Lucia	ESP		U Madrid		Original	Laboratory	Aetiology		Dairy
51	Subclinical mastitis in ewes and its effect on lamb performance	1992	J Anim Sci	Keisler	3	Moffatt	USA		U Missouri		Original	Field	Effects		Meat
52	Number and evaluation of somatic cells in the milk of goats and sheep	1992	Archiv Lebensmittelhygiene	Hahn	6	Heeschen	GER		Institute of Hygiene Germany		Original	Field	Diagnosis		Dairy
53	The descriptive epidemiology of udder lesions in Northern Iraqi ewes	1992	Prev Vet Med	Sulaiman	2	Alsadi	IRQ		U Mosul		Original	Field	Epidemiology		Dairy
54	Hydrophobicity of ruminant mastitis Staphylococcus aureus	1992	Curr Microbiol	Baselga	6	Amorena	ESP		SIA DGA-Zaragoza		Original	Laboratory	Aetiology		Dairy
55	Use of the Fossomatic method to determine somatic cell counts	1993	J Dairy Sci	Gonzalo	4	San Primitivo	ESP		U Leon		Original	Field	Diagnosis		Dairy
56	Experimental intramammary infection of ewes with Staphylococcus aureus subsp. anaerobius	1993	Res Vet Sci	Delafuente	5	Suarez	ESP		U Madrid		Original	Experimental	Pathogenesis		Dairy
57	Role of an intramammary device protection against experimentally induced staphylococcal mastitis in ewes	1993	Am J Vet Res	Penades	6	Amorena	ESP		SIA DGA-Zaragoza		Original	Experimental	Control		Dairy
58	Differentiation of staphylococci from ewe and goat milk samples	1993	Deut Tierarzt Wochen	Deinhofer	2	Pernhaner	AUT		U Vienna		Original	Field	Aetiology		Dairy
59	Phase variation of slime production in staphylococcus aureus -implications in colonisation and virulence	1993	Infect Immun	Baselga	6	Amorena	ESP		SIA DGA-Zaragoza		Original	Experimental	Aetiology	Pathogenesis	Dairy
60	Adherence of ruminant mastitis Staph aureus strains to epithelial cells from ovine mammary gland primary cultures and from a rat intestinal cell line	1993	Vet Microbiol	Iturralde	4	Amorena	ESP		U Zaragoza	SIA DGA -Zaragoza	Original	Laboratory	Pathogenesis		Dairy
61	Genetic and environmental causes of variation in mastitis in sheep	1993	Small Rumin Res	Larsgard	2	Vaabenoe	NOR		Agricultural U Norway		Original	Field	Risk factors		Meat
62	Immunobiological and biochemical properties of mutants	1994	J Immunol	Murray	9	Schlievert	USA		U Minnesota		Original	Laboratory	Aetiology		Dairy
63	Some properties of coagulase negative staphylococci isolated from cases of ovine mastitis	1994	Epidemiol Infect	Fthenakis	4	Jones	GBR		U London		Original	Laboratory	Aetiology		Meat
64	Use of liposome immunopotentiated exopolysaccharide as a component of an ovine mastitis staphylococcal vaccine	1994	Vaccine	Amorena	3	Albizu	ESP		SIA DGA-Zaragoza		Original	Experimental	Control		Dairy

65	Prevalence and aetiology of subclinical mastitis in ewes of Southern Greece	1994	Small Rumin Res	Fthenakis	1		GRE		Lapapharm		Original	Field	Epidemiology		Dairy
66	Factors influencing variation of test day milk-yield, somatic cell count, fat and protein in dairy sheep	1994	J Dairy Sci	Gonzalo	4	Primitivo	ESP		U Leon		Original	Field	Diagnosis	Effects	Dairy
67	Etiology and prevalence of subclinical mastitis in the Manchega sheep at mid-late lactation	1994	Small Rumin Res	Delacruz	8	Amorena	ESP		SIA DGA-Zaragoza		Original	Field	Aetiology	Epidemiology	Dairy
68	Genetic parameters of test day measures for somatic cell counts, milk yield and protein percentage of milking ewes	1994	J Dairy Sci	Baro	3	Sanprimitivo	ESP	GBR	U Leon	U Edinburgh	Original	Field	Effects	Diagnosis	Dairy
69	Milk losses caused by chronical mastitis	1995	Wiener Tierarzt Monats	Deutz	4	Fuchs	AUT		U Vienna		Original	Field	Effects		Dairy
70	Effect of variable traction on the teatcup during machine milking of ewes with or without hand stripping	1995	Annal Zoot	Peris	5	Rodriguez	ESP		U Valencia		Original	Field	Pathogenesis	Effects	Dairy
71	Examination of systems that exert traction on the teatcup and reduce teat bending in machine-milking of ewes	1995	Annal Zoot	Peris	5	Torres	ESP		U Valencia		Original	Field	Pathogenesis	Effects	Dairy
72	California mastitis test and Whiteside test in diagnosis of subclinical mastitis of dairy ewes	1995	Small Rumin Res	Fthenakis	1		GRE		Greek Army Centre Biological Research Greece		Original	Field	Diagnosis		Dairy
73	Variation in somatic cell counts in ewes milk during lactation	1995	Zivocisna Vyroba	Margetin	5	Foltys	SLK		Research Institute in Animal Production Slovakia		Original	Field	Diagnosis		Dairy
74	Use of somatic cell counts for the detection of subclinical mastitis in sheep	1995	Small Rumin Res	Mavrogenis	4	Taliotis	CYP		Agricultural Research Institute Cyprus	Ministry of Agriculture Cyprus	Original	Field	Diagnosis		Dairy
75	Frequency of subclinical mastitis and observations on somatic cell counts in ewes milk in Northern Greece	1995	Anim Sci	Stefanakis	4	Samartzi	GRE		Aristotle U Thessaloniki		Original	Field	Epidemiology	Diagnosis	Dairy
76	The distibution of Pasteurella haemolytica serotypes among cattle, sheep, and goats in South Africa and their association with disease	1995	Onderstep J Vet Res	Odendaal	2	Henton	ZAR		Onderstepoort Veterinary Institute South Africa		Original	Laboratory	Aetiology		Meat
77	Relationship between somatic cell count and intramammary infection of the half udder in dairy ewes	1995	J Dairy Sci	Gonzalez-Rodriguez	4	Carmenes	ESP		U Leon		Original	Field	Diagnosis		Dairy
78	Coagulase negative staphylococci as a pathogen of subclinical and clinical mastitis in three flocks of milk sheep	1996	Tierarzt Umsch	Winter	2	Hofer	AUT		U Vienna	Disease Institute Austria	Original	Field	Aetiology		Meat
79	Somatic cell counts in milk of Welsh Mountain, Dorset-Horn and Chios ewes throughout lactation	1996	Small Rumin Res	Fthenakis	1		GRE		Lapapharm		Original	Field	Diagnosis		Dairy
80	Mastitis in ewes	1996	Comp Cont Edu Pract Vet	Kirk	1		USA		U California Davis	U Auburn	Review				
81	Total and differential cell count in milk of primiparous Comisana ewes without clinical signs of mastitis	1996	Small Rumin Res	Morgante	5	Duranti	ITL		Inst Semeiotica Medica e Metodologia Clinica Veterinaria	Istituto di Zootecnica Generale	Original	Field	Diagnosis		Dairy
82	Evaluation of the California mastitis test as a discriminant method to detect subclnical mastitis in ewes	1996	Small Rumin Res	Gonzalez-Rodriguez	2	Carmenes	ESP		U Leon		Original	Field	Diagnosis		Dairy
83	Resistance to antibiotics in Staphylococcus aureus at ewe masitis, in sheep milk and its products	1996	Veterinarni Medicina	Simko	2	Bartko	SLK		U Kosice	Ministry of Agriculture Slovakia	Original	Laboratory	Treatment		Dairy
84	Cytokine induced inflammation in the ovine teat and udder	1996	Vet Immunol Immunopathol	Persson	5	Seow	SWE		U Uppsala	CSIRO	Original	Experimental	Pathogenesis		Meat
85	Mastitis in a flock of milking sheep	1996	Small Rumin Res	Kirk	3	Maas	USA		U Auburn	U California Davis	Original	Field	Aetiology	Epidemiology	Dairy
86	Total and differential cell count by direct microscopic method on ewe milk	1996	J Vet Med A	Morgante	4	Beghelli	ITL		U Perugia		Original	Laboratory	Diagnosis		Dairy
87	Subclinical mastitis in sheep	1996	Ind Vet J	Rao	3	Sreemannarayana	IND		Veterinary Polyclinic		Original	Field	Aetiology	Epidemiology	Dairy
88	Somatic cells in sheep milk in relation to milk production and composition during sucking and milking	1996	Zivocisna Vyroba	Margetin	4	Foltys	SLK		Vyskumny Ustav Zivocisnej Vyroby		Original	Field	Effects		Dairy
89	Accumulation of leucocytes and cytokines in the lactating ovine udder during mastitis due to Staphylococcus aureus and Escherichia coli	1997	Res Vet Sci	Persson-Waller	3	Seow	SWE	AUS	U Uppsala	CSIRO	Original	Experimental	Pathogenesis		Meat
90	Experimental studies on Pasteurella mastitis in ewes	1997	Large Anim Pract	Al-Ani	3	Karim	JOD	IRQ	Jordan U Science Technology	U Baghdad	Original	Experimental	Pathogenesis		Dairy
91	Evaluation of the Staph-Zym system in the identification of Staphylococci	1997	J Basic Microbiol	Burriel	2	Scott	GBR		U London		Original	Laboratory	Aetiology		Meat
92	Leukocyte and cytokine accumulation in the ovine teat and udder during endotoxin-induced inflammation	1997	Vet Res Comm	Waller	4	Seow	SWE	AUS	U Uppsala	CSIRO	Original	Experimental	Pathogenesis		Meat
93	Udder orf infection and its role in ovine clinical mastitis caused by Pasteurella haemolytica	1997	J Trace Elem Med Biol	Burriel	1		GBR		U London		Original	Field	Risk factors		Meat
94	Dynamics of intramammary infection in the sheep caused by coagulase-negative staphylococci and its influence on udder tissue and milk composition	1997	Vet Rec	Burriel	1		GBR		U London		Original	Field	Pathogenesis		Meat
95	Milk differential cell counts in relation to total counts in Sardinian ewes	1997	Small Rumin Res	Cuccuru	6	Contini	ITL		U Sassari		Original	Field	Diagnosis		Dairy
96	Serological diagnosis of bovine, caprine and ovine mastitis caused by Listeria monocytogenes by using an enzyme-linked immunosorbent assay	1997	J Clin Microbiol	Bourry	3	Poutrel	FRA		INRA		Original	Laboratory	Diagnosis		Dairy
97	Comparison of different machine milking clusters on dairy ewes with large size teats	1997	Annal Zoot	Fernandez	7	Torres	ESP		U Valencia		Original	Experimental	Risk factors		Dairy
98	Resistance of coagulase negative staphylococci isolated from sheep to various antimicrobial agents	1997	Res Vet Sci	Burriel	1		GBR		U London		Original	Laboratory	Treatment		Meat
99	Leukotoxic factors produced by staphylococci of ovine origin	1997	Microbiol Res	Burriel	2	Dagnall	GBR		U London		Original	Laboratory	Treatment		Meat
100	Corynebacterium mastitidis sp. nov., isolated from milk of sheep with subclinical mastitis	1997	Int J Syst Bacteriol	Fernandez-Garayzabal	7	Dominguez	ESP		U Madrid		Original	Field	Aetiology		Dairy
101	Pharmacokinetics and penetration of danofloxacin from the blood into the milk of ewes	1997	Vet Res	Shem-Tov	4	Saran	ISR		Ministry of Agriculture Israel		Original	Laboratory	Treatment		Dairy
102	Isolation of Pasteurella haemolytica from grass, drinking water and straw bedding	1997	Curr Microbiol	Burriel	1		GBR		U London		Original	Field	Aetiology		Meat
103	Electron microscopic study of milk sediments -Qualitative and quantitative observations	1997	APMIS	Burriel	2	Brendle	GBR		U London		Original	Laboratory	Pathogenesis		Meat
104	Pharmacokinetics and penetration of marbofloxacin from blood into the milk of cows and ewes	1997	J Vet Med A	Shem-Tov	4	Saran	ISR		Ministry of Agriculture Israel		Original	Laboratory	Treatment		Dairy
105	Serum and milk iron levels during sheep intramammary infection caused by coagulase-negative staphylococci	1997	Biolog Trace Elem Res	Burriel	2	Heys	GBR		U London		Original	Field	Risk factors		Meat
106	In vitro presence of capsular polysaccharide in coagulase-negative staphylococci of ovine origin	1997	Microbiologica	Burriel	1		GBR		U London		Original	Laboratory	Aetiology		Meat
107	Epidemiological studies of clinical and subclinical ovine mastitis in Awassi sheep in northern Jordan	1998	Prev Vet Med	Lafi	4	Alawah	JOD		Jordan U Science Technology		Original	Field	Epidemiology	Aetiology	Dairy
108	The effects of inoculation of Listeria monocytogenes into the ovine mammary gland	1998	Vet Microbiol	Tzora	3	Linde	GRE	GER	TEI Epirus	U Leipzig	Original	Experimental	Pathogenesis		Dairy
109	Histopathological and ultrastructural observation of ovine mammary glands experimentally inoculated with coagulase-negative staphylococci	1998	Zblatt Vetmed B	Burriel	1		GBR		U London		Original	Laboratory	Pathogenesis		Meat
110	Isolation of coagulase-negative staphylococci from the milk and environment of sheep	1998	J Dairy Res	Burriel	1		GBR		U London		Original	Field	Aetiology	Pathogenesis	Meat
111	A comparison of methods used in species identification of coagulase negative staphylococci isolated from the milk of sheep	1998	Vet J	Burriel	2	Scott	GBR		U London		Original	Laboratory	Aetiology		Meat
112	Bovine and ovine mastitis in Dhuleil Valley of Jordan	1998	Veterinar Arhiv	Lafi	2	Hailat	JOD		Jordan U Science Technology		Original	Field	Aetiology	Epidemiology	Dairy
113	Susceptibility to antibiotics of staphylococcal isolates from cases of ovine or bovine mastitis	1998	Small Rumin Res	Fthenakis	1		GRE		Lapapharm		Original	Laboratory	Treatment		Dairy
114	Corynebacterium camporealensis associated with subclinical mastitis in sheep	1998	Int J Syst Bacteriol	Fernandez-Garayzabal	6	Dominguez	ESP		U Madrid		Original	Laboratory	Aetiology		Dairy
115	Expression of surface antigens of blood and mammary leukocytes in lactating and dry ewes	1998	Vet Immunol Immunopathol	Persson-Waller	2	Colditz	SWE	AUS	U Uppsala	CSIRO	Original	Laboratory	Pathogenesis		Meat
116	The carriage of Pasteurella haemolytica in sheep and its transfer between ewes and lambs	1998	J Comp Pathol	Scott	2	Jones	GBR		U London		Original	Field	Aetiology	Pathogenesis	Meat
117	Mastitis in dairy ewes associated with Serratia macrescens	1998	Small Rumin Res	Tzora	2	Fthenakis	GRE		TEI Epirus	Lapapharm	Original	Field	Aetiology		Dairy
118	Effects of long-term recombinant bovine somatotropin (bST) administration on milk yield, milk composition and mammary gland health of dairy ewes	1998	Small Rumin Res	Brozos	5	Tsakalof	GRE		Aristotle U Thessaloniki		Original	Field	Risk factors		Dairy
119	Classification of the clinical types of udder disease affecting nursing ewes	1998	Small Rumin Res	Calavas	4	Sulpice	FRA		Centre Ecopathologie Animale		Original	Field	Epidemiology		Dairy
120	Mammary and systemic aspergillosis in dairy sheep	1998	Vet Pathol	Perez	5	Jensen	ESP	DEN	U Leon	U Copenhagen	Original	Field	Aetiology		Dairy
121	Detection of Staphylococcus aureus in milk by use of polymerase chain reaction analysis	1998	Am J Vet Res	Khan	8	Baek	USA		U Illinois		Original	Laboratory	Aetiology		Meat
122	Milk composition, somatic cell count, clinical and bacteriological investigation	1998	Prakt Tierarzt	Wittek	3	Beck	GER		U Liepzig		Original	Field	Aetiology	Diagnosis	Dairy
123	Use of benzethine cloxacillin for control of ovine mastitis	1998	Ind Vet J	Sheikh	2	Wilayat	IND		U Kashmir		Original	Field	Treatment		Dairy
124	Streptococcus parasanguinis: new pathogen associated with asymptomatic mastitis in sheep	1998	Emerg Inf Dis	Fernandez-Garayzabal	6	Dominguez	ESP		U Madrid		Original	Laboratory	Aetiology		Meat
125	Heritability of test day somatic cell counts and its relationship with milk yield and protein percentage in dairy ewes	1998	J Dairy Sci	El-Saied	3	San Primitivo	ESP	EGY	U Leon	U Cairo	Original	Field	Risk factors	Diagnosis	Dairy
126	Naturally occuring subclinical ovine mastitis associated with Listeria monocytogenes	1998	Small Rumin Res	Fthenakis	4	Linde	GRE	GER	U Thessaly	U Leipzig	Original	Field	Aetiology		Dairy
127	Incidence risk and aetiology of mammary abnormalities in dry ewes in 10 flocks in Southern Greece	1998	Prev Vet Med	Saratsis	5	Fthenakis	GRE		Aristotle U Thessaloniki	U Thessaly	Original	Field	Aetiology	Epidemiology	Dairy
128	The influence of ewe intramammary infection caused by coagulase negative staphylococci on the milk constituents total protein, albumin, potassium and sodium	1998	Anim Sci	Burriel	2	Wagstaff	GBR		U London		Original	Field	Effects		Meat
129	Adhesion of coagulase positive and coagulase negative staphylococci	1999	Small Rumin Res	Burriel	1		GBR		U London		Original	Laboratory	Pathogenesis		Meat
130	Prevalence and aetiology of subclinical mastitis in the Madrid region	1999	Small Rumin Res	Las Heras	3	Fernandez-Garayzabal	ESP		U Madrid		Original	Field	Aetiology	Epidemiology	Dairy
131	Genetic parameters of lactation cell counts and milk and protein yield in dairy ewes	1999	J Dairy Sci	El-Saied	4	San Primitivo	ESP	EGY	U Leon	U Cairo	Original	Field	Risk factors		Dairy

132	Effect of administration of vitamin E and selenium during the dry period	1999	J Dairy Sci	Morgante	6	Ranucci	ITL		Inst Semeiotica Medica e Metodologia Clinica Veterinaria		Original	Field	Control		Dairy
133	The intramammary inflammatory response of genetically resistant Merino ewes infected with Haemonchus contortus	1999	Int J Parasitol	Thamsborg	5	Lea	DEN	AUS	U Copenhagen	U New England	Original	Experimental	Pathogenesis		Meat
134	The effect of experimentally induced subclinical mastitis on the milk yield of dairy ewes	1999	Small Rumin Res	Saratsis	4	Fthenakis	GRE		Aristotle U Thessaloniki	U Thessaly	Original	Field	Effects		Dairy
135	The effect of experimental infectious mastitis on leukocyte subpopulations and cytokine production in non-lactating ewes	1999	Zblatt Vetmed B	Persson-Waller	2	Colditz	SWE	AUS	U Uppsala	CSIRO	Original	Experimental	Pathogenesis		Meat
136	Resistance patterns of ovine mastitis pathogens	1999	Berl Munch Tierarzt Wochs	Winter	3	Baumgartner	AUT		U Vienna	Vet P'I	Original	Laboratory	Treatment		Meat
137	Effect of intramammary infusion of beta-1,3-glucan or interleukin-2 on leukocyte subpopulations in mammary glands of sheep	1999	Am J Vet Res	Waller	2	Colditz	SWE	AUS	U Uppsala	CSIRO	Original	Experimental	Pathogenesis		Meat
138	Outbreak of acute ovine mastitis associated with Pseudomonas aeruginosa infection	1999	Vet Rec	Las Heras	4	Fernandez-Garayzabal	ESP		U Madrid		Original	Field	Aetiology		Dairy
139	Effect of stocking density on ewes milk yield, udder health and microenvironment	1999	J Dairy Res	Sevi	5	Muscio	ITL		U Foggia		Original	Field	Risk factors		Dairy
140	Somatic cell counts determined by the Coulter or Fossomatic counter and their relationship to administration of oxytocin	2000	Small Rumin Res	Burriel	1		GBR		U London		Original	Laboratory	Diagnosis		Meat
141	Extensive fibrinous pleurisy associated with Streptococcus dysgalactiae	2000	Vet Rec	Scott	1		GBR		U Edinburgh		Original	Field	Effects		Meat
142	The effect of tilmicosin administered to ewes prior to lambing on incidence of clinical mastitis	2000	Can Vet J	Croft	6	Dick	CDN		U Guelph	Provel	Original	Field	Control		Meat
143	Effects of parity on milk yield composition, somatic cell count, renneting parameters and bacteria counts of Comisana ewes	2000	Small Rumin Res	Sevi	5	Annicchiarico	ITL		U Foggia	Inst Sperimentale per la Zootecnia Foggia	Original	Field	Epidemiology		Dairy
144	Intramammary Aspergillus fumigatus infection in dairy ewes associated with antibiotic dry therapy	2000	Vet Rec	Las Heras	8	Fernandez-Garayzabal	ESP		U Madrid		Original	Field	Risk factors		Dairy
145	Field evaluation of flunixin meglumine in the supportive treatment of ovine mastitis	2000	J Vet Pharmacol Ther	Fthenakis	1		GRE		U Thessaly		Original	Field	Treatment		Dairy
146	Quality of ewe milk as affected by stocking density and litter treatment with bentonite	2000	Ital J Food Sci	Sevi	6	Dell'Aquila	ITL		U Foggia	Inst Sperimentale per la Zootecnia Foggia	Original	Field	Epidemiology		Dairy
147	Antimicrobial susceptibility of corynebacteria isolated from ewe's mastitis	2001	Int J Antimicrob Ag	Fernandez	6	Moreno	ESP		U Madrid		Original	Laboratory	Treatment		Dairy
148	Lack of association between retention of fetal membranes and mastitis in ewes	2001	Vet Rec	Leontides	3	Tzora	GRE		U Thessaly	TEI Epirus	Original	Field	Epidemiology		Dairy
149	Effect of slime on adherence of Staphylococcus aureus isolated from bovine and ovine mastitis	2001	Vet Microbiol	Aguilar	3	Iturralde	ESP		U Zaragoza	Ministry of Agriculture Spain	Original	Laboratory	Aetiology	Pathogenesis	Dairy
150	Udder disease etiology, milk somatic cell counts and NAGase activity in Israeli Assaf sheep throughout lactation	2001	Small Rumin Res	Leitner	8	Saran	ISR		Ministry of Agriculture Israel		Original	Field	Aetiology	Diagnosis	Dairy
151	The role of coagulase negative Staphycoccus spp and associated somatic cell counts	2001	J Dairy Sci	Pengov	1		SLV		U Ljubljana		Original	Field	Aetiology	Diagnosis	Dairy
152	Outbreak of subclinical mastitis in a flock of dairy sheep associated with Burkholderia cepacia complex infection	2001	J Clin Microbiol	Berratua	8	Govan	ESP	GBR	NEIKER	U Edinburgh	Original	Field	Aetiology		Dairy
153	Mastitis in ewes	2001	In Pract	Winter	1		GBR		U Liverpool		Review				
154	Relationships among somatic cell count, California mastitis test, impedance and bacteriological status of milk in goats and sheep in early lactation	2001	Small Rumin Res	McDougall	6	Scruton	NZL	USA	Animal Health Centre	Ministry of Agriculture USA	Original	Field	Aetiology	Diagnosis	Meat
155	Mastitis of sheep and goats	2001	Vet Clin NA Food Anim Pract	Menzies	2	Ramanoon	CDN		U Guelph		Review				
156	Relationship between mammary gland infection and milk immune parameters in Sardinian breed ewes	2001	Small Rumin Res	Moroni	2	Cuccuri	ITL		U Milan	U Sassari	Original	Field	Pathogenesis	Diagnosis	Dairy
157	Genetic analysis for mastitis resistance and milk somatic cell score in French Lacaune dairy sheep	2001	Gen Sel Evol	Barrillet	5	Jacquin	FRA		INRA		Original	Field	Risk factors		Dairy
158	Binding of a surface protein of Staphylococcus aureus to cultured ovine mammary gland epithelial cells	2001	Vet Microbiol	Aguillar	2	Iturralde	ESP		U Zaragoza	SIA DGA-Zaragoza	Original	Experimental	Pathogenesis		Dairy
159	Airspace effects on the yield and quality of ewe milk	2001	J Dairy Sci	Sevi	5	Muscio	ITL		U Foggia		Original	Field	Risk factors		Dairy
160	Udder health status of dairy flocks during one lactation period with special regards to infections caused by coagulase-negative staphylococci	2002	Wiener Tierarzt Monats	Winter	3	Baumgartner	AUT		U Vienna	Vet P'I	Original	Field	Aetiology		Meat
161	Physiological range of milk contents and somatic cell count in milk of ewes with special regard to urea	2002	Tierarzt Prakt Aus Gross Nutz	Flock	5	Baumgartner	AUT		U Vienna		Original	Field	Diagnosis		Dairy
162	The effect of stage of lactation on milk quantity and number of somatic cells in sheep milk	2002	MilchWissenSchaft	Antunac	5	Samarzjia	CRO		U Zagreb		Original	Field	Risk factors		Dairy
163	Prevalence and etiology of subclinical mastitis in intensively managed flocks and related changes in the yield and quality of ewe milk	2002	Small Rumin Res	Albenzio	4	Sevi	ITL		U Foggia		Original	Field	Aetiology	Risk factors	Dairy
164	Unusual outbreak of clinical mastitis in dairy sheep caused by Streptococcus equi subsp zooepidemicus	2002	J Clin Microbiol	Las Heras	6	Fernandez-Garayzabal	ESP		U Madrid		Original	Field	Aetiology		Dairy
165	Effect of infectious status and parity on somatic cell count and California mastitis test in pampinta dairy ewes	2002	J Vet Med B	Suarez	6	Canavesio	ARG		Inst Nacional Tecnologico Agropecuaria		Original	Field	Diagnosis		Meat
166	Mammary pathogens and their relationship to somatic cell count	2002	J Dairy Sci	Gonzalo	4	San Primitivo	ESP		U Leon		Original	Field	Aetiology	Diagnosis	Dairy
167	Microbiological quantity and somatic cell count of ewe milk with special reference	2002	J Dairy Sci	Ariznabarreta	3	Carriedo	ESP		U Leon		Original	Field	Aetiology		Dairy
168	Production and novel quantification of haemolysins produced by coagulase-negative staphylococci isolated from subclinical mastitis in sheep	2002	Microbiologica	Kanellos	2	Burriel	GBR		U London		Original	Laboratory	Aetiology		Meat
169	Immunological responses of the lactating ovine udder following experimental challenge with Staphylococcus epidermidis	2002	Vet Immunol Immunopathol	Winter	2	Colditz	AUT	AUS	U Vienna	CSIRO	Original	Experimental	Pathogenesis		Meat
170	Phagocytic capacity of leucocytes in sheep mammary secretions following weaning	2002	J Anat	Tatarczuch	4	Lee	AUS		U Melbourne		Original	Experimental	Pathogenesis		Meat
171	Physicochemical, microbiological and coagulating properties of ewe's milk produced on the Calabrian Mount Poro plateau	2002	Int J Dairy Technol	Micari	5	Cufari	ITL		U Reggio Calabria		Original	Field	Diagnosis		Dairy
172	DNA macrorestriction analysis by pulsed-field gel electrophoresis of Pseudomonas aeruginosa isolates from mastitis in dairy sheep	2002	Vet Rec	Las Heras	6	Fernandez-Garayzabal	ESP		U Madrid		Original	Field	Aetiology		Dairy
173	Antibody responses in sheep vaccinated against Staphylococcus aureus mastitis: a comparison of two experimental vaccines containing different adjuvants	2002	Vet Res Comm	Tollersrud	6	Lund	NOR		National Veterinary Institute Norway	Agricultural U Norway	Original	Experimental	Control		Meat
174	Efficacy of dry-off treatment in sheep	2003	Small Rumin Res	Chaffer	7	Saran	ISR		Kimron Veterinary Institute		Original	Field	Control		Dairy
175	Therapeutic efficacy of tilmicosin in ovine mammary infections	2003	Small Rumin Res	Nacari	5	De Montis	ITL		U Messina	U Sassari	Original	Experimental	Treatment		Dairy
176	Methicillin resistance in staphylococci isolated from subclinical mastitis in sheep	2003	Microbiologica	Corrente	4	Ventriglia	ITL		U Bari		Original	Laboratory	Treatment		Dairy
177	New advances in epizootiology and control of ewe mastitis	2003	Liv Prod Sci	Bergonier	2	Berthelot	FRA		Ecole Nationale Veterinaire de Toulouse	INRA	Review				
178	Influence of pulsation rate on udder health and teat thickness changes in dairy ewes	2003	J Dairy Sci	Peris	5	Fernandez	ESP		U Valencia		Original	Field	Risk factors		Dairy
179	Period prevalence and etiology of subclinical mastitis in Awassi sheep in southern Jordan	2003	Small Rumin Res	Al-Majali	2	Jawabreh	JOD		Jordan U Science Technology	Ministry of Agriculture Jordan	Original	Field	Aetiology	Epidemiology	Dairy
180	Leucotoxic activities of Staphylococcus aureus strains isolated from cows, ewes, and goats with mastitis: importance of LukM/LukF'-PV leukotoxin	2003	Clin Diagn Lab Immunol	Rainard	5	Poutrel	FRA		INRA		Original	Laboratory	Aetiology		Dairy
181	Genetic parameters for milk somatic cell scores and relationships with production traits in French Lacaune dairy sheep	2003	J Dairy Sci	Rupp	4	Barillet	FRA		INRA		Original	Field	Risk factors		Dairy
182	Effects of litter management on airborne particulates in sheep in sheep houses and on the yield and quality of ewe milk	2003	Liv Prod Sci	Sevi	5	Centoducati	ITL		U Foggia	U Bari	Original	Field	Risk factors		Dairy
183	Dynamics of experimentally induced Staphylococcus epidermidis mastitis in East Friesian milk ewes	2003	J Dairy Sci	Winter	4	Colditz	AUT	AUS	U Vienna	CSIRO	Original	Experimental	Pathogenesis		Meat
184	Evaluation of diagnostic procedures for subclinical mastitis in meat-producing sheep	2003	J Dairy Res	Clements	3	Fitzpatrick	GBR		U Glasgow	U London	Original	Field	Diagnosis		Meat
185	Serum amyloid A in the serum and milk mastitis of ewes with mastitis induced experimentally with Staphylococcus epidermidis	2003	Vet Rec	Winter	3	Walshe	AUT	AUS	U Vienna	CSIRO	Original	Experimental	Pathogenesis	Diagnosis	Meat
186	Immune response, udder health and productive traits of machine milked and suckling ewes	2003	Small Rumin Res	Albenzio	6	Sevi	ITL		U Foggia		Original	Experimental	Risk factors	Pathogenesis	Dairy
187	Udder infection and milk somatic cell count, NAGase activity and milk composition-fat, protein and lactose-in Israeli-Assaf and Awassi sheep	2003	Small Rumin Res	Leitner	8	Saran	ISR		Kimron Veterinary Institute		Original	Field	Diagnosis	Effects	Dairy
188	Genetic parameters of somatic cell count in dairy sheep considering the type of mammary pathogen effect	2003	J Anim Breed Gen	Gonzalo	6	San Primitivo	ESP		U Leon		Original	Field	Risk factors		Dairy
189	Mastitis of dairy small ruminants	2003	Vet Res	Bergonier	5	Berthelot	FRA		Ecole Nationale Veterinaire de Toulouse		Review				
190	Study on frequency, etiology and some enzymatic activities of subclinical ovine mastitis in Urmia, Iran	2003	Small Rumin Res	Batavani	4	Dawoodi	IRN		U Urmia		Original	Field	Aetiology	Epidemiology	Dairy
191	Clinical and ultrasonographic findings in the mammary gland of sheep	2003	NZ Vet J	Franz	4	Baumgartner	AUT		U Vienna		Original	Field	Diagnosis		Meat
192	Antimicrobial drug susceptibility of Staphylococcus aureus strains isolated from bovine and ovine mammary glands	2003	J Dairy Sci	Pengov	2	Ceru	SLV		U Ljubljana		Original	Field	Treatment		Dairy
193	Hard ewe's milk cheese manufactured from milk of three different groups of somatic cell counts	2003	J Dairy Sci	Jaeggi	7	Wendorff	USA		U Wisconsin		Original	Field	Effects		Dairy
194	Genotyping of Staphylococcus aureus from various sites on farms with dairy sheep using pulsed-field gel electrophoresis	2003	Vet Microbiol	Vautor	5	Pepin	FRA		AFSSA		Original	Laboratory	Aetiology		Dairy
195	Genetic analysis of somatic cell count and milk traits in Manchega ewes -Mean lactation and test-day approaches	2003	Liv Prod Sci	Serrano	4	Jurado	ESP		INIA		Original	Field	Risk factors		Dairy
196	A case of sporadic ovine mastitis caused by Listeria monocytogenes and its effect on contamination of raw milk and raw-milk cheeses produced in the on-farm dairy	2003	J Dairy Res	Schoder	5	Wagner	AUT		U Vienna		Original	Field	Aetiology		Dairy
197	Influence of vacuum level and overmilking on udder health and teat thickness changes in dairy ewes	2003	J Dairy Sci	Peris	6	Fernandez	ESP		U Valencia		Original	Field	Risk factors		Dairy
198	Physical examination of the mammary gland and milk indicators of udder health in East Friesian milk sheep	2004	Tierarzt Prax	Fahr	4	Al-Hammoud	GER		Paul Lister Institute		Original	Field	Diagnosis		Meat

199	Interrelationships between the activities of the plasmin system in goats and sheep experiencing subclinical mastitis, casein degradation and milk yield	2004	S Afr Anim J Sci	Silanikove	4	Merin	ISR		Agri Research Organisation Israel			Original	Field	Pathogenesis		Dairy
200	Changes in milk composition as affected by subclinical mastitis	2004	S Afr Anim J Sci	Merin	5	Leitner	ISR		Agri Research Organisation Israel	Kimron Veterinary Institute		Original	Field	Effects		Dairy
201	Factors influencing milk quantity and quality in Assaf sheep and goat crossbreds	2004	S Afr Anim J Sci	Leitner	7	Saran	ISR		Kimron Veterinary Institute			Original	Field	Effects		Dairy
202	Clinical and histopathological aspects of naturally occurring mastitis caused by Listeria monocytogenes in cattle and ewes	2004	J Vet Med B	Winter	7	Wagner	AUT		U Vienna			Original	Field	Aetiology	Pathogenesis	Dairy
203	Changes in milk composition as affected by subclinical mastitis in sheep	2004	J Dairy Sci	Leitner	8	Silanikove	ISR		Agri Research Organisation Israel	Kimron Veterinary Institute		Original	Field	Effects		Dairy
204	Experimentally induced teat stenosis in dairy ewes: clinical, pathological and ultrasonographic features	2004	J Comp Pathol	Mavrogianni	6	Taitzoglou	GRE		U Thessaly	Aristotle U Thessaloniki		Original	Experimental	Pathogenesis		Dairy
205	Evaluation of molecular and immunological techniques for the diagnosis of mammary aspergillosis in ewes	2004	Vet Microbiol	Garcia	5	Blanco	ESP		U Madrid			Original	Laboratory	Diagnosis		Dairy
206	Effects of selective and complete dry therapy on prevalence of intramammary infection and on milk yield in the subsequent lactation in dairy ewes	2004	J Dairy Res	Gonzalo	4	San Primitivo	ESP		U Leon			Original	Field	Control		Dairy
207	In vitro adherence and invasion of ovine mammary epithelium by Mannheimia (Pasteurella) haemolytica	2004	Vet J	Vilela	3	Morgan	GBR	POR	U Bristol	U Lisbon		Original	Experimental	Pathogenesis		Meat
208	Effects of lambing season and stage of lactation on ewe milk quality	2004	Small Rumin Res	Sevi	5	Muscio	ITL		U Foggia			Original	Field	Risk factors		Dairy
209	Effects of somatic cell count and stage of lactation on the plasmin activity and cheese-making properties of ewe milk	2004	J Dairy Sci	Albenzio	6	Sevi	ITL		U Foggia			Original	Field	Effects		Dairy
210	Antibiotic resistance and epidemiological typing of Staphylococcus aureus strains from ovine and rabbit mastitis	2004	Int J Antimicrob Ag	Goni	6	Gomez	ESP		U Zaragoza			Original	Laboratory	Aetiology	Treatment	Dairy
211	Case report: high prevalence of ovine mastitis, caused by coagulase-negative staphylococci and predisposed by increased gossypol consumption	2004	Small Rumin Res	Fthenakis	5	Tzora	GRE		U Thessaly	TEI Epirus		Original	Field	Epidemiology	Risk factors	Dairy
212	Phenotypic and genotypic characteristics of Staphylococcus aureus isolates from raw bulk-tank milk samples of goats and sheep	2004	Vet Microbiol	Scherrer	5	Stephan	SWI		U Zurich			Original	Laboratory	Aetiology		Dairy
213	Bacteriology and somatic cell counts in milk samples from ewes on a Scottish farm	2004	Can J Vet Res	Hariharan	4	Keefe	CDN		U Prince Edward Island	Moredun Research Institute		Original	Field	Aetiology	Diagnosis	Meat
214	Streptococcus equi subsp ruminatorum subsp nov., isolated from mastitis in small ruminants	2004	Int J Syst Evol Microbiol	Fernandez	7	Fernandez-Garayzabal	ESP		U Madrid			Original	Laboratory	Aetiology		Dairy
215	The effects of inoculation of Mannheimia haemolytica into the teat of lactating ewes	2005	Vet Res	Mavrogianni	8	Saratsis	GRE		U Thessaly	Aristotle U Thessaloniki		Original	Experimental	Pathogenesis		Dairy
216	Investigation of antioxidant enzymes and some biochemical parameters in ewes with gangrenous mastitis	2005	Turk J Vet Anim Sci	Cetin	5	Korkmaz	TUR		U Harran			Original	Field	Diagnosis		Dairy
217	Phenotypic and genetic analysis of udder health using SCC in Valle del Belice dairy sheep	2005	Ital J Anim Sci	Portolano	5	Giaccone	ITL		U Palermo			Original	Field	Risk factors		Dairy
218	Evaluation of a combined vaccine against staphylococcal mastitis in ewes	2005	Bull Vet Inst Pulawy	Hadimli	4	Sayin	TUR		U Selcuk			Original	Field	Control		Dairy
219	Somatic cells in sheep milk	2005	Med Wet	Olechnowicz	2	Jaskowski	POL		U Krakow			Original	Field	Diagnosis		Dairy
220	Factors of variation influencing bulk tank somatic cell count in dairy sheep	2005	J Dairy Sci	Gonzalo	7	San Primitivo	ESP		U Leon			Original	Field	Risk factors		Dairy
221	Genetic diversity of Staphylococcus aureus isolated from ovine intramammary infections in Norway	2005	Vet Microbiol	Mork	5	Waage	NOR		National Veterinary Institute Norway	U Oslo		Original	Laboratory	Aetiology		Meat
222	Studies on the use of Blu-Gard spray	2005	Small Rumin Res	Klingmair	3	Baumgartner	AUT		U Vienna			Original	Field	Control		Dairy
223	Investigation and control of mastitis outbreaks caused by Pseudomonas aeruginosa in a sheep flock and a goat	2005	Berl Munch Tierarzt Wochs	Yeruham	7	Goshen	ISR		U Jerusalem			Original	Field	Aetiology	Control	Dairy
224	Genetic parameters of udder traits, somatic cell score, and milk yield in Latxa sheep	2005	J Dairy Sci	Legarra	2	Ugarte	ESP		NEIKER			Original	Field	Risk factors		Dairy
225	Characterization of 26 isolates of Staphylococcus aureus, predominantly from dairy sheep, using four different techniques of molecular epidemiology	2005	J Vet Diagn Invest	Vautor	6	Pepin	FRA		AFSSA			Original	Laboratory	Aetiology		Dairy
226	Comparison of Staphylococcus aureus genotypes recovered from cases of bovine, ovine, and caprine mastitis	2005	J Clin Microbiol	Mork	5	Waage	NOR		National Veterinary Institute Norway	U Oslo		Original	Laboratory	Aetiology		Dairy
227	Relationship between welfare and udder health indicators in dairy ewes	2006	Vet Res Comm	Caroprese	4	Sevi	ITL		U Foggia			Original	Field	Risk factors		Dairy
228	Teat disorders predispose ewes to clinical mastitis	2006	Vet Res	Mavrogianni	5	Fthenakis	GRE		U Thessaly			Original	Experimental	Pathogenesis		Dairy
229	Toll-like receptor gene polymorphism and its relationship with somatic cell concentration and natural bacterial infections of the mammary gland in sheep	2006	Folia Microbiol	Swiderek	6	Mikula	POL		U Warsaw	U Kosice	Academy of Sciences Slovakia	Original	Laboratory	Risk factors		Dairy
230	Relationship between blood lymphocyte phenotype, DRB1 (MHC class II) gene polymorphism and somatic cell count in ewe milk	2006	Bull Vet Inst Pulawy	Swiderek	4	Gruszcynska	POL		U Warsaw			Original	Field	Pathogenesis		Dairy
231	Use of somatic cell counts and California Mastitis Test results from udder halves milk samples to detect subclinical intramammary infection in Awassi sheep	2006	Small Rumin Res	Lafi	1		JOD		Jordan U Science Technology			Original	Field	Diagnosis		Dairy
232	Physiological and pathological threshold of somatic cell counts in ewe milk	2006	Small Rumin Res	Berthelot	5	Bergonier	FRA		Ecole Nationale Veterinaire de Toulouse			Original	Field	Diagnosis		Dairy
233	Relationship between somatic cell counts and the properties of yoghurt made from ewes' milk	2006	Int Dairy J	Vivar-Quintana	3	Revilla	ESP		U Salamanca			Original	Field	Effects		Dairy
234	Effects of hand milking on the bacterial flora of the mammary gland and teat duct	2006	J Dairy Res	Mavrogianni	5	Fthenakis	GRE		U Thessaly			Original	Field	Risk factors		Dairy
235	Description and validation of a novel technique to study the bacterial flora of the teat duct of ewes	2006	Small Rumin Res	Mavrogianni	3	Fthenakis	GRE		U Thessaly			Original	Field	Diagnosis		Dairy
236	Molecular typing of Staphylococcus aureus isolated from cows, goats and sheep with intramammary infections on the basis of gene polymorphisms and toxins genes	2006	j Vet Med B	Vermecati	7	Moroni	ITL		U Milan			Original	Laboratory	Aetiology		Dairy
237	The potential of measuring serum amyloid A in individual ewe milk and in farm bulk milk for monitoring udder health on sheep dairy farms	2006	Res Vet Sci	Winter	4	Baumgartner	AUT		U Vienna			Original	Field	Pathogenesis	Diagnosis	Dairy
238	Effects of machine-milking on the bacterial flora of teat duct and mammary gland of ewes	2006	J Vet Med B	Skoufos	4	Tzora	GRE		TEI Epirus			Original	Field	Pathogenesis		Dairy
239	Clinical aspects and characteristics of the milk in sheep with mastitis	2007	Pesq Vet Brazil	Santos	4	Simao	BRA		U Pernambuco			Original	Experimental	Pathogenesis		Dairy
240	Detection of quantitative trait loci influencing somatic cell score in Spanish Churra sheep	2007	J Dairy Sci	Gutierrez-Gil	7	Arranz	ESP		U Leon			Original	Field	Risk factors		Dairy
241	Relationship between beta lactoglobulin and subclinical mastitis in Valle del Belice sheep breed	2007	Ital J Anim Sci	Gigli	6	Maizon	ITL		U Palermo			Original	Field	Risk factors		Dairy
242	Effect of weather conditions on somatic cell score in Sicilian Valle del Belice ewes	2007	Ital J Anim Sci	Finocchiaro	3	Portolano	ITL		U Palermo			Original	Field	Risk factors		Dairy
243	Drug resistance in isolated bacteria from milk of sheep and goats with subclinical mastitis	2007	Iran J Vet Res	Ebrahimi	3	Karimi	IRN		U Shahrekord			Original	Laboratory	Treatment		Dairy
244	Mastitis in small ruminants	2007	Small Rumin Res	Contreras	7	Gonzalo	ESP		U Murcia	U Leon		Review				
245	Somatic cells of goat and sheep milk: analytical, sanitary, productive and technological aspects	2007	Small Rumin Res	Raynal-Ljutovac	4	Gonzalo	FRA	ESP	Inst Technologie Produits Laitiers Caprins	U Leon		Review				
246	Monitoring goat and sheep milk somatic cell counts	2007	Small Rumin Res	Paape	8	Miller	USA		Ministry of Agriculture USA			Review				
247	Economic weights of somatic cell score in dairy sheep	2007	Animal	Leggara	5	Arranz	ESP		NEIKER			Original	Field	Effects		Dairy
248	Genetic parameters for milk somatic cell score and relationships with production traits in primiparous dairy sheep	2007	J Dairy Sci	Riggio	5	Bovenhuis	ITL	NED	U Palermo	U Wageningen		Original	Field	Risk factors		Dairy
249	Udder health and somatic cell counts in ewes	2007	Agrarforschung	Maurer	2	Schaeren	SWI		Forsch Sanstalt Agroscope Liebefeld Posieux			Original	Field	Diagnosis		Dairy
250	The effect of machine or hand milking on milk production, composition and SCC	2007	Small Rumin Res	Sinapis	1		GRE		Aristotle U Thessaloniki			Original	Field	Diagnosis	Effects	Dairy
251	Clinical, bacteriological, cytological and pathological features of teat disorders in ewes	2007	J Vet Med A	Mavrogianni	2	Fthenakis	GRE		U Thessaly			Original	Field	Risk factors		Dairy
252	Bacterial flora and risk of infection of the ovine teat duct and mammary gland throughout lactation	2007	Prev Vet Med	Mavrogianni	3	Fthenakis	GRE		U Thessaly			Original	Field	Risk factors		Dairy
253	Genetics of disease resistance in sheep and goats	2007	Small Rumin Res	Bishop	2	Morris	GBR	NZL	Roslin Institute	AgResearch New Zealand		Review				
254	Characterization of Staphylococcus aureus isolates from buffalo, bovine, ovine, and caprine milk samples collected in Rio de Janeiro State, Brazil	2007	Appl Environ Microbiol	Aires-De-Sousa	6	de Lencastre	POR	USA	New U Lisbon	U Rockfeller		Original	Laboratory	Aetiology		Dairy
255	Presence of subepithelial lymphoid nodules in the teat of ewes	2007	Anat Histol Embryol	Mavrogianni	5	Fthenakis	GRE		U Thessaly			Original	Laboratory	Pathogenesis		Dairy
256	The bacterial flora in the teat duct of ewes can protect against and can cause mastitis	2007	Vet Res	Fragkou	5	Fthenakis	GRE		U Thessaly			Original	Experimental	Pathogenesis		Dairy
257	Presence of sub-epithelial lymphoid tissues in the teat of ewe-lambs and adult ewes	2007	Small Rumin Res	Fragkou	7	Fthenakis	GRE		U Thessaly			Original	Laboratory	Pathogenesis		Dairy
258	Effect of dietary zinc upon in vitro bacterial adherence to ovine mammary epithelium	2007	Rev Med Vet	Saianda	5	Vilela	POR		U Lisbon			Original	Laboratory	Pathogenesis		Dairy
259	Time-to-event analysis of mastitis at first-lactation in Valle del Belice ewes	2007	Liv Sci	Portolano	5	Maizon	ITL		U Palermo			Original	Field	Risk factors		Dairy
260	Influence of somatic cell count and breed on capillary electrophoretic protein profiles of ewes' milk: a chemometric study	2007	J Dairy Sci	Rodriguez-Nogales	3	Revilla	ESP		U Salamanca	U Valladoliv		Original	Field	Effects		Dairy
261	Differences in susceptibility to Mannheimia haemolytica-associated mastitis	2007	J Dairy Res	Fragkou	8	Fthenakis	GRE		U Thessaly			Original	Experimental	Pathogenesis		Dairy
262	Effect of intramammary infection in Bergamasca meat sheep on milk parameters and lamb growth	2007	J Dairy Res	Moroni	4	Boettcher	ITL		U Milan	Institute of Agricultural Biology and Biotechnology		Original	Field	Effects		Dairy
263	Clinical mastitis in ewes; bacteriology, epidemiology and clinical features	2007	Acta Vet Scand	Mork	5	Sviland	NOR		National Veterinary Institute Norway			Original	Field	Aetiology		Dairy
264	Characterization of Staphylococcus aureus isolates recovered from dairy sheep farms (agr group, adherence, slime, resistance to antibiotics)	2007	Small Rumin Res	Vautor	6	Dellamonica	FRA		AFSSA	Archat Hospital		Original	Laboratory	Aetiology		Dairy
265	Study of the leucocytic formula of milk in the ewes of race Ouled-Djellal in the east of Algeria	2007	Afr J Agr Res	Laouabdia-Sellami	3	Guetarni	ALG		U Badji Mokhtar	U Blida		Original	Field	Diagnosis		Dairy

266	Pseudomonas aeruginosa mastitis outbreaks in sheep and goat flocks	2007	Vet Immunol Immunopathol	Leitner	2	Krifucks	ISR			Kimron Veterinary Institute			Original	Experimental	Control		Dairy
267	Program for the control of subclinical mastitis in dairy Chios breed ewes during lactation	2007	Small Rumin Res	Kiossis	4	Boscos	GRE			Aristotle U Thessaloniki			Original	Field	Control		Dairy
268	Phenotypic and genotypic characterization of Pseudomonas aeruginosa strains isolated from mastitis	2007	J Dairy Res	Sela	6	Leitner	ISR			Kimron Veterinary Institute	Agri Research Organisation Israel		Original	Laboratory	Aetiology		Dairy
269	Teat lesions predispose to invasion of the ovine mammary gland by Mannheimia haemolytica	2007	J Comp Pathol	Fragkou	5	Fthenakis	GRE			U Thessaly			Original	Experimental	Risk factors	Pathogenesis	Dairy
270	Studies on mastitis in sheep, caused by Coxiella burnetii	2007	Biotechnol Biotecholog Equip	Martinov	1		BUL			National Veterinary Institute Bulgaria			Original	Field	Aetiology		Dairy
271	Patterns of maternal-offspring behaviour of dairy sheep and potential association with mammary health	2007	Can J Anim Sci	Gougoulis	9	Fthenakis	GRE			U Thessaly			Original	Field	Pathogenesis		Dairy
272	Order of sheep entry into the milking parlour and its relationship with their milkability	2007	Appl Anim Behav Sci	Villagra	5	Fernandez	ESP			U Valencia			Original	Field	Risk factors		Dairy
273	Profile and evolution of antimicrobial resistance of ovine mastitis pathogens (1995-2004)	2008	Small Rumin Res	Lollai	6	Leori	ITL			Inst Zooprofilatico Sardegna	National Reference Centre Sheep & Goats		Original	Laboratory	Treatment		Dairy
274	Estimate of milk and curd yield loss of sheep and goats with intrammamary infection and its relation to somatic cell count	2008	Small Rumin Res	Leitner	3	Merin	ISR			Kimron Veterinary Institute	Agri Research Organisation Israel		Original	Field	Effects		Dairy
275	Dry-off treatment of Assaf sheep: efficacy as a management tool for improving milk quantity and quality	2008	Small Rumin Res	Shwimmer	6	Leitner	ISR			Kimron Veterinary Institute	Ministry of Agriculture Israel		Original	Field	Control		Dairy
276	Disease threats to sheep associated with intensification of pastoral farming	2008	NZ Vet J	Ridler	1		GBR			U London			Review				
277	Ovine Listeria monocytogenes mastitis and human exposure via fresh cheese from raw milk -the impact of farm management, milking and cheese manufacturing practices	2008	MilchWissenSchaft	Schoder	6	Wagner	AUT			U Vienna			Original	Experimental	Effects		Dairy
278	Subclinical mastitis in sheep: etiology, epidemiology and diagnosis methods	2008	Kaf Univ Vet Fakult Derg	Yagci	1		TUR			U Kirikkale			Review				
279	Antimicrobial use in the Alberta sheep industry	2008	Can J Vet Res	Avery	7	McEwen	CDN			Public Health Agency Canada	Ministry of Agriculture Canada		Original	Field	Treatment		Dairy
280	Effects of lamb sucking on the bacterial flora of teat duct and mammary gland of ewes	2008	Reprod Dom Anim	Gougoulis	6	Fthenakis	GRE			U Thessaly			Original	Experimental	Pathogenesis		Dairy
281	Breeding for resistance to mastitis in United Kingdom sheep, a review and economic appraisal	2008	Vet Rec	Conington	4	Bunger	GBR			Scottish Agricultural College			Review				
282	Subclinical mastitis changes the patterns of maternal-offspring behaviour in dairy sheep	2008	Vet J	Gougoulis	6	Fthenakis	GRE			U Thessaly			Original	Experimental	Effects		Dairy
283	EC and CMT detect subclinical mastitis in dairy sheep but less sensitive than in dairy cows	2008	Landbauforschung Volkenrode	Barth	2	Knappstein	GER			Federal Research Inst For Rural areas Germany	Max Ruber Inst		Original	Field	Diagnosis		Dairy
284	Effects of experimental challenge of ewes with Mannheimia haemolytica on subsequent milk composition	2008	J Dairy Res	Fragkou	12	Fthenakis	GRE			U Thessaly			Original	Experimental	Effects		Dairy
285	Evaluation of a prophylactic method against clinical mastitis in Santa Ines ewes	2008	Arq Brasil Med Vet Zoot	Melo	6	Mata	BRA			U Brazilia	Agricultural Research Organisation Brasil		Original	Field	Control		Dairy
286	Cellular and microbiological profile of Santa Ines ewes in the lactation and the post-weaning period.	2008	Pesq Vet Brazil	Blagitz	11	Della Libera	BRA			U Sao Paolo			Original	Field	Diagnosis		Dairy
287	Influence of an intramammary infusion at drying-off of combined penethamate hydriodide, benethamine penicillin, and framycetin sulfate on intramammary infections and somatic cell counts in dairy sheep	2008	J Dairy Sci	Linage	2	Gonzalo	ESP			U Leon			Original	Field	Control		Dairy
288	Risk factors and impacts of clinical and subclinical mastitis in commercial meat-producing sheep flocks in Quebec, Canada	2008	Prev Vet Med	Arsenault	4	Belanger	CDN			U Montreal			Original	Field	Risk factors	Effects	Meat
289	Individual animal risk factors for clinical mastitis in meat sheep in Norway	2008	Prev Vet Med	Waage	2	Vatn	NOR			U Oslo	Ministry of Agriculture Norway		Original	Field	Risk factors		Meat
290	Prevalence and etiology of subclinical mastitis in Awassi dairy ewes in southern Turkey	2009	Turk J Vet Anim Sci	Ergun	8	Demir	TUR			U Mustafa Kemal			Original	Field	Aetiology	Epidemiology	Dairy
291	Ultrasonographic examination of the ovine udder	2009	Tierarzt Prakt Aus Gross Nutz	Hiepler	3	Wehrend	GER			U Giessen			Original	Experimental	Diagnosis		Meat
292	Estimation of (co)variance components of nematode parasites resistance and somatic cell count in dairy sheep	2009	Ital J Anim Sci	Sechi	7	Casu	ITL			AGRIS Sardegna			Original	Field	Risk factors		Dairy
293	Study of beta-defensin polymorphisms in Valle del Belice dairy sheep	2009	Ital J Anim Sci	Monteleone	4	Portolano	ITL			U Palermo			Original	Field	Risk factors		Dairy
294	Usefulness of the E-test for the determination of the susceptibility of Staphylococcus sp. Isolated from milk of sheep and goats with subclinical mastitis to amikacin and amoxicillin-clavulanic acid	2009	Bull Vet Inst Pulawy	Kirecci	4	Saribay	TUR			U Ataturk	U Mustafa Kemal		Original	Laboratory	Treatment		Dairy
295	The effect of estrus synchronization treatments on somatic cell count of transitional-anestrus Awassi ewes' milk	2009	Trop Anim Hlth and Prod	Talafha	3	Ababneh	JOD			Jordan U Science Technology			Original	Field	Risk factors		Dairy
296	Response to somatic cell count-based selection for mastitis resistance in a divergent selection experiment in sheep	2009	J Dairy Sci	Rupp	7	Foucras	FRA			INRA	U Toulouse		Original	Experimental	Risk factors		Dairy
297	Pseudomonas spp. isolated in dairy herds. A new protocol with PCR real time by using "DUAL FRET" probes for ovine mastitis	2009	Large Anim Rev	Orru	9	Orru	ITL			U Cagliari	Inst Zooprofilatico Sardegna		Original	Field	Diagnosis		Dairy
298	Risks of antibiotic residues in milk following intramammary and intramuscular treatments in dairy sheep	2009	Analyt Chim Acta	Pengov	2	Kirbis	SLV			U Ljubljana			Original	Field	Treatment		Dairy
299	Protection from Staphylococcus aureus mastitis associated with poly-N-acetyl beta-1,6 glucosamine specific antibody production using biofilm-embedded bacteria	2009	Vaccine	Perez	16	Amorena	ESP			U Navara	SIA DGA-Zaragoza	U Zaragoza	Original	Experimental	Control		Dairy
300	Simultaneous lack of catalase and beta-toxin in Staphylococcus aureus leads to increased intracellular survival in macrophages and epithelial cells and to attenuated virulence in murine and ovine models	2009	Microbiol SGM	Martinez-Pulgarin	4	de la Fuente	ESP			U Madrid			Original	Laboratory	Pathogenesis		Dairy
301	Association between a case study of asymptomatic ovine listerial mastitis and the contamination of soft cheese and cheese processing environment with Listeria monocytogenes in Portugal	2009	Foodborne Pathog Dis	Pintado	6	McLauchlin	POR			U Lisbon	Ministry of Health UK		Original	Field	Effects		Dairy
302	Pharmacokinetics and efficacy of teicoplanin against intramammary infections in sheep	2009	Vet Rec	Naccari	8	Naccari	ITL			U Messina	U Milan		Original	Field	Treatment		Dairy
303	Lamb mortality in an outbreak of Yersinia pseudotuberculosis mastitis, as a collateral effect of colostrum feeding for Lentivirus-control	2009	Small Rumin Res	Juste	5	de Heredia	ESP			NEIKER			Original	Field	Aetiology	Effects	Dairy
304	Development of serological proteome analysis of mastitis by Staphylococcus aureus in ewes	2009	J Microbiol Meth	Le Marechal	8	Le Loir	FRA			INRA			Original	Laboratory	Aetiology		Dairy
305	Difference in virulence between Staphylococcus aureus isolates causing gangrenous mastitis versus subclinical mastitis in a dairy sheep flock	2009	Vet Res	Vautor	8	Lindsay	FRA	GBR		AFSSA	U London		Original	Field	Pathogenesis		Dairy
306	Molecular typing of Staphylococcus aureus strains from ovine mastitis by pulsed-field gel electrophoresis and polymerase chain reaction based on coagulase and protein A gene polymorphisms	2009	J Vet Diagn Invest	Ciftci	5	Sogut	TUR			U Ondokuz Mayis	U Amasya		Original	Laboratory	Aetiology		Dairy
307	Effect of somatic cell count level on functional longevity in Valle del Belice dairy sheep assessed using survival analysis	2009	J Dairy Sci	Riggio	5	van Arendonk	ITL	NED		U Palermo	U Wageningen		Original	Field	Diagnosis		Dairy
308	Endemic contagious agalactia in sheep and goats: clinical evaluation, treatment and vaccination	2010	J Anim Vet Adv	Macun	6	Kalin	TUR			U Kirikkale	U Firat		Original	Field	Treatment		Dairy
309	Clinical findings in sheep farms affected by recurrent bacterial mastitis	2010	Small Rumin Res	Marogna	5	Leori	ITL			Inst Zooprofilatico Sardegna			Original	Field	Aetiology		Dairy
310	Phenotypic and genetic relationships between udder morphology and udder health in dairy ewes	2010	Small Rumin Res	Casu	4	Carta	ITL			U Sassari			Original	Field	Risk factors		Dairy
311	The role of lactate dehydrogenase, alkaline phosphatase and aspartate aminotransferase in the diagnosis of subclinical intramammary infections in dairy sheep and goats	2010	J Dairy Res	Katsoulos	6	Kritas	GRE			U Thessaly	Aristotle U Thessaloniki		Original	Field	Diagnosis		Dairy
312	The induction of lymphoid follicle-like structures in the ovine teat duct following experimental infection with Mannheimia haemolytica	2010	Vet J	Fragkou	12	Fthenakis	GRE			U Thessaly			Original	Experimental	Pathogenesisi		Dairy
313	Proteinogram and serum concentrations of copper, iron and zinc in Santa Ines ewes with Staphylococcus aureus experimentally induced mastitis.	2010	Pesq Vet Brazil	Costa	8	de Mendonca	BRA			U Pernambuco			Original	Experimental	Pathogenesis	Diagnosis	Dairy
314	Management and sanitary practices in ewe dairy farms and bulk milk somatic cell count	2010	Spanish J Agr Res	Molina	5	Molina	ESP			U Castilla La Mancha	U Valencia		Original	Field	Risk factors	Diagnosis	Dairy
315	Genotypic characterization of coagulase-negative staphylococci Isolated from sheep milk in Slovakia	2010	Acta Vet Brno	Pilipcincova	4	Travnicek	SLK			U Kosice			Original	Laboratory	Aetiology		Dairy
316	Genetic parameters for somatic cell score according to udder infection status in Valle del Belice dairy sheep and impact of imperfect diagnosis of infection	2010	Gen Sel Evol	Riggio	4	Bishop	ITL	NED	GBR	U Palermo	U Wageningen	U Edinburgh	Original	Field	Risk factors		Dairy
317	Influences of somatic cell count on milk composition and dairy farm profitability	2010	Int J Dairy Technol	El-Tahawy	2	El-Far	EGY			U Alexandria			Original	Field	Effects		Dairy
318	Identification of coagulase-negative staphylococci isolated from ovine milk samples by PCR-RFLP of 16S rRNA and gap genes	2010	Vet Microbiol	Onni	7	Tolla	ITL			Inst Zooprofilatico Sardegna			Original	Laboratory	Aetiology		Dairy
319	Small ruminant mastitis in Brazil.	2010	Pesq Vet Brazil	Peixoto	3	da Costa	BRA			U Vale Sao Francisco			Original	Field	Aetiology		Dairy
320	Etiology and profile of antimicrobial sensitivity of bacteria from small ruminant mastitis and relationship of diagnostic techniques.	2010	Pesq Vet Brazil	Peixoto	5	da Costa	BRA			U Vale Sao Francisco			Original	Laboratory	Treatment		Dairy
321	Mannheimia species associated with ovine mastitis	2010	J Clin Microbiol	Omaleki	4	Browning	AUS			U Melbourne			Original	Laboratory	Aetiology		Meat
322	Acute mastitis induces upregulation of expression of plasminogen activator-related genes by blood monocytes and neutrophils in dairy ewes	2010	Vet Immunol Immunopathol	Theodorou	6	Politis	GRE			Agricultural U Athens			Original	Laboratory	Pathogenesis		Dairy
323	Staphylococcus aureus mastitis in Texel sheep associated with suckling twins	2010	Vet Rec	Koop	3	Rieterse	NED			U Utrecht			Original	Field	Risk factors		Meat
324	Etiology and antimicrobial susceptibilities of bacteria isolated from sheep with mastitis in northeastern Para, Brazil.	2010	Pesq Vet Brazil	Silva	8	Barbosa	BRA			U Para			Original	Laboratory	Aetiology	Treatment	Dairy
325	Antimicrobial susceptibility of coagulase-negative staphylococci isolated from meat-producing ewes with mastitis	2010	Arq Brasil Med Vet Zoot	Della Libera	8	Gomes	BRA			U Sao Paolo			Original	Laboratory	Treatment		Dairy
326	Effect of freezing and preincubaton on isolation of coagulase-negative staphylococci from ewes' milk samples	2011	Semina Cien Agrar	Blagitz	7	Della Libera	BRA			U Sao Paolo			Original	Laboratory	Aetiology		Dairy
327	Effects of intramammary infections on somatic cell score and milk yield in Sarda sheep	2011	NZ Vet J	Cuccuru	7	Bronzo	ITL			U Milan	U Sassari		Original	Field	Effects		Dairy
328	Sheep with scrapie and mastitis transmit infectious prions through the milk	2011	J Virol	Ligios	11	Sigurdson	ITL	SWI	USA	Inst Zooprofilatico Sardegna	U Zurich	U California Davis	Original	Field	Effects		Meat
329	Rates and determinants of antimicrobial use, including extra-label, on Ontario sheep farms	2011	Can J Vet Res	Moon	7	Menzies	CDN			U Guelph			Original	Field	Treatment		Dairy
330	Investigation of toxin genes of Staphylococcus aureus strains isolated from gangrenous mastitis in ewes	2011	Kaf Univ Vet Fakult Derg	Tel	5	Demir	TUR			U Harran	U Mustafa Kemal		Original	Laboratory	Aetiology		Dairy
331	Characterization of ovine mastitis in Santa Ines sheep in the north of Minas Gerais: occurrence, etiology and epidemiology	2011	Rev Caatinga	de Morais	6	Duarte	BRA			U Minas Gerais			Original	Field	Aetiology	Risk factors	Dairy
332	Antimicrobial resistance of Staphylococcus aureus and coagulase negative staphylococci isolated from mastitis milk samples from sheep and goats	2011	Schweizer Arch Tierarzt	Kuntz	6	Zweifel	SWI			U Zurich			Original	Laboratory	Treatment		Meat

333	Differential leucocyte count for ewe milk with low and high somatic cell count	2011	J Dairy Res	Albenzio	2	Caroprese	ITL			U Foggia			Original	Laboratory	Diagnosis		Dairy
334	Selenium, vitamin E and vitamin A blood concentrations in dairy sheep flocks with increased or low clinical mastitis incidence	2011	Small Rumin Res	Giadinis	7	Fthenakis	GRE			Aristotle U Thessaloniki	U Thessaly		Original	Field	Risk factors		Dairy
335	Staphylococcus aureus seroproteomes discriminate ruminant isolates causing mild or severe mastitis	2011	Vet Res	Le Marechal	15	Le Loir	FRA			INRA	ANSES	Agrocampus Ouest	Original	Laboratory	Pathogenesis		Dairy
336	Antimicrobial susceptibilities and population structure of Staphylococcus epidermidis associated with ovine mastitis	2011	Vet Microbiol	Onni	4	Tola	ITL			Inst Zooprofilatico Sardegna			Original	Laboratory	Aetiology	Treatment	Dairy
337	Transmission of Mannheimia haemolytica from the tonsils of lambs to the teat of ewes during sucking	2011	Vet Microbiol	Fragkou	8	Fthenakis	GRE			U Thessaly			Original	Experimental	Aetiology	Risk factors	Dairy
338	Principles of mastitis treatment in sheep and goats	2011	Vet Clin NA Food Anim Pract	Mavrogianni	4	Fthenakis	GRE			U Thessaly			Review				
339	Effects of glandular bacterial infection and stage of lactation on milk clotting parameters: comparison among cows, goats and sheep	2011	Int Dairy J	Leitner	3	Silanikove	ISR			Kimron Veterinary Institute	Agri Research Organisation Israel		Original	Field	Effects		Dairy
340	Influence of storage and preservation on microbiological quality of silo ovine milk	2011	J Dairy Sci	de Carnica	3	Gonzalo	ESP			U Leon			Original	Field	Aetiology	Risk factors	Dairy
341	Transcriptomic analysis of milk somatic cells in mastitis resistant and susceptible sheep upon challenge with Staphylococcus epidermidis and Staphylococcus aureus	2011	BMC Genomics	Bonnefont	11	Rupp	FRA			INRA	U Toulouse		Original	Laboratory	Risk factors		Dairy
342	Genome sequences of two Staphylococcus aureus ovine strains that induce severe (strain O11) and mild (strain O46) mastitis	2011	J Bacteriol	Le Marechal	10	Le Loir	FRA			INRA	ANSES	Agrocampus Ouest	Original	Laboratory	Aetiology	Pathogenesis	Dairy
343	Impact of intramammary antimicrobial dry treatment and teat sanitation on somatic cell count and intramammary infection in dairy ewes	2011	Small Rumin Res	Spanu	3	Ruegg	USA			U Wisconsin			Original	Field	Control		Dairy
344	Strengthening insights into host responses to mastitis infection in ruminants by combining heterogeneous microarray data sources	2011	BMC Genomics	Genini	25	Giuffra	ITL	USA	FRA	Parco Technologico Padano	U Pennsylvania	INRA	Original	Laboratory	Pathogenesis		Dairy
345	Novel sequence types (STs) of Staphylococcus aureus isolates causing clinical and subclinical mastitis in flocks of sheep in the northeast of Brazil	2011	J Dairy Res	de Almeida	4	Mamizuka	BRA			U Sao Paolo			Original	Laboratory	Aetiology	Pathogenesis	Dairy
346	Preliminary investigation into the effect of freezing and a cryopreservant on the recovery of mastitis pathogens from ewe milk	2011	J Dairy Sci	Smith	4	Green	GBR			U Warwick			Original	Laboratory	Aetiology		Meat
347	Cellular dynamics and microbiological quality of milk of Santa Ines ewes accompanied during lactation	2011	Pesq Vet Brazil	Guarana	8	De Mendonca	BRA			U Pernambuco			Original	Field	Diagnosis		Dairy
348	Effect of subclinical mastitis on proteolysis in ovine milk	2011	J Dairy Sci	Marti de Olives	5	Molina	ESP			U Miguel Hernandez	U Valencia		Original	Experimental	Effects		Dairy
349	Molecular basis of virulence in Staphylococcus aureus mastitis	2011	Plos One	Le Marechal	16	Loir	FRA			INRA	ANSES	Agrocampus Ouest	Original	Laboratory	Pathogenesis		Dairy
350	The role of Mannheimia species in ovine mastitis	2011	Vet Microbiol	Omaleki	4	Joanne	AUS			U Melbourne			Review				
351	Isolation of microbial pathogens of subclinical mastitis from raw sheep's milk of Epirus (Greece) and their role in its hygiene	2011	Anaerobe	Fotou	9	Demertzis	GRE			U Ioannina	TEI Epirus		Original	Field	Aetiology		Dairy
352	The Importance of staphylococci and threshold value of somatic cell count for diagnosis of sub-clinical mastitis in Pirlak sheep at mid-lactation	2011	Reprod Dom Anim	Ozenc	6	Dogan	TUR			U Afyon			Original	Field	Diagnosis		Dairy
353	Somatic cell count and California Mastitis Test as a diagnostic tool for subclinical mastitis in ewes	2012	Acta Scient Vet	Pradiee	10	Schmidt	BRA			U Rio Grande do Sul	U Pelotas		Original	Field	Diagnosis		Dairy
354	Antimicrobial susceptibility, presence of resistance genes and biofilm formation in coagulase-negative staphlococci isolated from subclinical sheep mastitis	2012	Kaf Univ Vet Fakult Derg	Ergun	6	Boyar	TUR			U Mustafa Kemal			Original	Laboratory	Aetiology	Diagnosis	Dairy
355	Identifying the bacteria causing ovine gangrenous mastitis and detection of Staphylococcus aureus in gangrenous milk by PCR	2012	Kaf Univ Vet Fakult Derg	Tel	2	Bozkaya	TUR			U Harran			Original	Field	Aetiology		Dairy
356	Identification and antibiotic sensitivity of the causative organisms of sub-clinical mastitis in sheep and goats	2012	Pak Vet J	Islam	7	Kim	KOR	BGD		U Shonbuk	Inst Livestock Research Bangladesh		Original	Field	Aetiology	Treatment	Dairy
357	Somatic cell counts and total bacterial counts in bulk tank of small ruminants	2012	Sloven Vet Res	Olechnowicz	2	Jaskowski	POL			U Poznan			Original	Field	Diagnosis		Dairy
358	Detection of stapylococcal enterotoxin, methicillin-resistant and Panton-Valentine leukocidin genes in coagulase-negative staphylococci isolated from cows and ewes with subclinical mastitis	2012	Trop Anim Hlth and Prod	Unal	2	Cinar	TUR			U Kirikkale			Original	Laboratory	Aetiology		Dairy
359	Panton-Valentine leukocidin and some exotoxins of Staphylococcus aureus and antimicrobial susceptibility profiles of staphylococci isolated from milks of small ruminants	2012	Trop Anim Hlth and Prod	Unal	6	Yildirim	TUR			U Kirikkale			Original	Laboratory	Aetiology		Dairy
360	Genetic susceptibility to S. aureus mastitis in sheep: differential expression of mammary epithelial cells in response to live bacteria or supernatant	2012	Physiolog Genom	Bonnefont	8	Foucras	FRA			U Toulouse	INRA		Original	Laboratory	Pathogenesis		Dairy
361	Lactation stage and udder health status of Santa Ines ewes	2012	Arq Brasil Med Vet Zoot	Blagitz	9	Della Libera	BRA			U Sao Paolo			Original	Field	Risk factors		Dairy
362	Clonal diversity of Staphylococcus aureus originating from the small ruminants goats and sheep	2012	Vet Microbiol	Porrero	6	Aarestrup	DEN	ESP		Technical U Denmark	U Madrid		Original	Laboratory	Aetiology		Dairy
363	Investigation of the antibiotic resistance and biofilm formation of Staphylococcus aureus strains isolated from gangrenous mastitis of ewes	2012	Acta Vet Hung	Tel	5	Demir	TUR			U Harran	U Mustafa Kemal		Original	Laboratory	Aetiology		Dairy
364	Detection of Mycobacterium avium subsp paratuberculosis in intestinal and mammary tissues and in lymph nodes of sheep with different techniques and its relationship with enteric lesions	2012	Small Rumin Res	Preziuso	2	Renzoni	ITL			U Camerino			Original	Field	Aetiology		Dairy
365	Staphylococcus aureus proteins differentially recognized by the ovine immune response in mastitis or nasal carriage	2012	Vet Microbiol	Seyffert	13	Le Loir	FRA	BRA		INRA	U Minas Gerais	Agrocampus Ouest	Original	Laboratory	Aetiology		Dairy
366	Effects of intra-mammary bacterial infection with coagulase negative staphylococci and stage of lactation on shedding of epithelial cells and infiltration of leukocytes into milk: Comparison among cows, goats and sheep	2012	Vet Immunol Immunopathol	Leitner	6	Silanikove	ISR			Kimron Veterinary Institute	Agri Research Organisation Israel		Original	Experimental	Pathogenesis		Dairy
367	Immune competence of the mammary gland as affected by somatic cell and pathogenic bacteria in ewes with subclinical mastitis	2012	J Dairy Sci	Albenzio	5	Ciliberti	ITL			U Foggia			Original	Experimental	Pathogenesis		Dairy
368	"Milk-drop syndrome of ewes": investigation of the causes in dairy sheep in Greece	2012	Small Rumin Res	Giadinis	8	Fthenakis	GRE			Aristotle U Thessaloniki	U Thessaly		Original	Field	Aetiology		Dairy
369	Molecular epidemiology of Mannheimia haemolytica and Mannheimia glucosida associated with ovine mastitis	2012	J Vet Diagn Invest	Omaleki	4	Barber	AUS			U Melbourne			Original	Laboratory	Aetiology		Meat
370	Antimicrobial resistance of Staphylococcus spp. from small ruminant mastitis in Brazil	2012	Pesq Vet Brazil	Franca	8	Costa	BRA			U Vale Sao Francisco			Original	Laboratory	Treatment		Dairy
371	Long-term study of environmental effects on test-day somatic cell count and milk yield in Manchega sheep	2012	Small Rumin Res	Arias	7	Perez-Guzman	ESP			U Leon	Council Agriculture and Environment Spain		Original	Field	Risk factors		Dairy
372	Subclinical mastitis caused by Staphylococcus coagulase negative in meet-producing sheep	2012	Arq Brasil Med Vet Zoot	Tejada	6	Timm	BRA			U Pelotas			Original	Field	Aetiology		Meat
373	A cohort study of the associations between udder conformation, milk somatic cell count, and lamb weight in suckler ewes	2012	J Dairy Sci	Huntley	4	Green	GBR			U Warwick			Original	Field	Effects		Meat
374	Somatic cell count in small ruminants: friend or foe?	2012	Small Rumin Res	Souza	6	Cerquiera	BRA			U Minas Gerais			Review				
375	Effects of drying-off procedure of ewes' udder, with intramammary antibiotic administration, in subsequent mammary infection and development of mastitis	2012	J Hell Vet Med Soc	Petridis	6	Fthenakis	GRE			U Thessaly			Original	Experimental	Control		Dairy
376	Tissue distribution of enrofloxacin after intramammary or simulated systemic administration in isolated perfused sheep udders	2012	Am J Vet Res	Cadenas	7	Vieitez	ESP			U Leon			Original	Experimental	Treatment		Dairy
377	Molecular characterization of enterotoxigenic and borderline oxacillin resistant Staphylococcus strains from ovine milk	2012	Food Microbiol	Perillo	6	Colombo	ITL			U Roma			Original	Laboratory	Aetiology		Dairy
378	Survey of accessory gene regulator (agr) groups and TSST-1 encoding gene (tst) in Staphylococcus aureus isolated from ewes with mastitis in the northwest of Iran	2013	Iran J Vet Res	Saei	3	Zadeh	IRN			U Urmia			Original	Laboratory	Aetiology		Dairy
379	Peripheral blood and milk leukocytes subsets of lactating Sarda ewes	2013	Ital J Anim Sci	Bonelli	7	Nicolussi	ITL			Inst Zooprofilatico Sardegna			Original	Field	Pathogenesis		Dairy
380	Comparative analysis of agr groups and virulence genes among subclinical and clinical mastitis Staphylococcus aureus isolates from sheep flocks of the Northeast of Brazil	2013	Braz J Microbiol	de Almeida	4	Mamizuka	BRA			U Sao Paolo			Original	Laboratory	Pathogenesis		Dairy
381	Phenotypic and molecular characterization of isolates of Staphylococcus spp. obtained from sheep milk Chapeco-SC	2013	Semina Cien Agrar	Agnol	8	Girardini	BRA			U Santa Catarina			Original	Laboratory	Aetiology		Dairy
382	Etiology of mastitis in native goats and sheeps born and raised in Brazilian semi-arid biom	2013	Med Vet Recife	Silva	9	Mota	BRA			U Pernambuco			Original	Field	Aetiology		Dairy
383	Estimation of genetic and phenotypic parameters for bacteriological status of the udder, somatic cell score, and milk yield in dairy sheep using a threshold animal model	2013	Liv Sci	Tolone	3	Portolano	ITL			U Palermo			Original	Field	Risk factors		Dairy
384	Relationship among specific bacterial counts and total bacterial and somatic cell counts and factors influencing their variation in ovine bulk tank milk	2013	J Dairy Sci	de Garnica	7	Gonzalo	ESP			U Leon			Original	Field	Risk factors	Diagnosis	Dairy
385	Comparative molecular analysis of ovine and bovine Streptococcus uberis isolates	2013	J Dairy Sci	Gilchrist	5	Fontaine	GBR			Moredun Research Institute			Original	Laboratory	Aetiology		Meat
386	Molecular screening of ovine mastitis in different breeds	2013	J Dairy Sci	Guerreiro	5	Duarte	POR			IBPeja	U Aveiro		Original	Field	Aetiology		Dairy
387	Effects of drying-off procedure of ewes' udder in subsequent mammary infection and development of mastitis	2013	Small Rumin Res	Petridis	10	Fthenakis	GRE			U Thessaly			Original	Experimental	Control		Dairy
388	Consequences of reduced vitamin A administration on mammary health of dairy ewes	2013	Small Rumin Res	Koutsoumpas	8	Karatzias	GRE			Aristotle U Thessaloniki			Original	Field	Risk factors		Dairy
389	Outbreak of mastitis in sheep caused by multi-drug resistant Enterococcus faecalis in Sardinia, Italy	2013	Epidemiol Infect	Sanciu	6	Rappelli	ITL			U Sassari			Original	Field	Aetiology		Dairy
390	Methodology variations in milk somatic cell count from Santa Ines ewes	2013	Cienc Rural	Blagitz	7	Della Libera	BRA			U Sao Paolo			Original	Field	Diagnosis		Dairy
391	Ovine subclinical mastitis: Proteomic analysis of whey and milk fat globules unveils putative diagnostic biomarkers in milk	2013	J Proteom	Chiaradia	10	Benda	ITL			U Perugia	National Research Council Italy		Original	Field	Pathogenesis		Dairy
392	Staphylococcus aureus proteins differentially produced in ewe gangrenous mastitis or ewe milk	2013	Vet Microbiol	Le Marechal	9	Even	FRA			INRA	ANSES	Agrocampus Ouest	Original	Laboratory	Pathogenesis		Dairy
393	Receiver-operating characteristic curves for somatic cell scores and California mastitis test in Valle del Belice dairy sheep	2013	Vet J	Riggio	4	Portolano	ITL	NED		U Palermo	U Wageningen		Original	Field	Diagnosis		Dairy
394	Mastitis detection in sheep by infrared thermography	2013	Res Vet Sci	Martins	7	McManus	BRA			U Rio Grande do Sul			Original	Field	Diagnosis		Dairy
395	Proteolysis and partial dephosphorylation of casein are affected by high somatic cell counts in sheep milk	2013	Food Res Inter	Pinto	9	Chianese	ITL			U Napoli			Original	Field	Effects		Dairy
396	Production and release of antimicrobial and immune defense proteins by mammary epithelial cells following Streptococcus uberis infection of sheep	2013	Infect Immun	Addis	10	Schianchi	ITL			Ponto Conte Research	U Sassari		Original	Field	Pathogenesis		Dairy
397	Energy, protein and mineral metabolism in Santa Ines ewes, both healthy and with subclinical mastitis	2013	Pesq Vet Brazil	da Silva	6	de Mendonca	BRA			U Pernambuco			Original	Field	Effects		Dairy
398	Staphylococcus aureus and Escherichia coli prevalence in ovine bulk tank milk	2013	Small Rumin Res	de Garnica	5	Gonzalo	ESP			U Leon			Original	Field	Aetiology		Dairy
399	Study on the possible survival of Staphylococcus chromogenes through the dry period in dairy ewes	2013	Small Rumin Res	Kiossis	6	Boscos	GRE			Aristotle U Thessaloniki			Original	Field	Pathogenesis		Dairy

400	Analysis of somatic cell counts and risk factors associated with occurrence of bacteria in ewes of different primary purposes	2013	Liv Sci	Kern	4	Krieter	GER		U Kiel			Original	Field	Diagnosis		Dairy	Meat
401	Use of milk amyloid A in the diagnosis of subclinical mastitis in dairy ewes	2013	J Dairy Res	Miglio	7	Maresca	ITL		U Perugia	Inst Zooprofilattico Umbria e Marche		Original	Field	Diagnosis		Dairy	
402	Quantification of milk yield and composition changes as affected by subclinical mastitis during the current lactation in sheep	2013	J Dairy Sci	Marti de Olives	4	Peris	ESP		U Miguel Hernandez	U Valencia		Original	Experimental	Effects		Dairy	
403	Clinical findings related to intramammary infections in meat-producing ewes	2014	Trop Anim Hlth and Prod	Blagitz	8	Della Libera	BRA		U Sao Paolo			Original	Field	Aetiology		Meat	
404	Risk factors, etiology and clinical aspects of mastitis in meat ewes of Parana, Brazil	2014	Pesq Vet Brazil	Pereira	5	Lisboa	BRA		U Londrina			Original	Field	Aetiology	Risk factors	Meat	
405	Prevalence, pathogenic capability, virulence genes, biofilm formation, and antibiotic resistance of Listeria in goat and sheep milk confirms need of hygienic milking conditions	2014	Pathogens Global Health	Osman	4	Orabi	EGY		U Cairo			Original	Laboratory	Aetiology		Dairy	
406	Genetic parameters for somatic cell count, logSCC and somatic cell score of breeds: improved Valachian, Tsigai, Lacaune and their crosses	2014	Acta Vet Beograd	Makovicky	5	Diabelkova	SLV		U Komarno	U Pavol Jozef Safarik		Original	Field	Risk factors	Diagnosis	Dairy	
407	Trematode infections in pregnant ewes can predispose to mastitis during the subsequent lactation period	2014	Res Vet Sci	Mavrogianni	9	Fthenakis	GRE		U Thessaly			Original	Field	Risk factors		Dairy	
408	Mastitis in small ruminants	2014	Med Wet	Olechnowicz	2	Jaskowski	POL		U Poznan			Review					
409	Administration of antibiotics to ewes at the beginning of the dry-period	2014	J Dairy Res	Petridis	2	Fthenakis	GRE		U Thessaly			Review					
410	Thermographic variation of the udder of dairy ewes in early lactation and following an Escherichia coli endotoxin intramammary challenge in late lactation	2014	J Dairy Sci	Castro-Costa	6	Aguilo	ESP		U Barcelona			Original	Field	Diagnosis		Dairy	
411	Diagnosis of clinical or subclinical mastitis in ewes	2014	Small Rumin Res	Fragkou	3	Fthenakis	GRE		U Thessaly	Agricultural Organisation Dimitra Greece		Review					
412	The quality of Valle del Belice sheep's milk and cheese produced in the hot summer season in Sicily	2014	Dairy Sci Technol	Todaro	3	Scatassa	ITL		U Palermo	Inst Zooprofilatico Sicilia		Original	Field	Effects		Dairy	
413	Study on Correlation of Maedi-Visna Virus (MVV) with Ovine Subclinical Mastitis in Iran	2014	Ind J Microbiol	Asadpour	4	Jafari	IRN		U Tabriz			Original	Field	Risk factors		Dairy	
414	Ewe characteristics associated with neonatal loss in Norwegian sheep	2014	Prev Vet Med	Holmoy	3	Grohn	NOR	USA	U Life Science Norway	U Cornell		Original	Field	Effects		Meat	
415	Associations of pre-lambing body condition score and serum beta-hydroxybutyric acid and non-esterified fatty acids concentrations with periparturient health of Chios dairy ewes	2014	Small Rumin Res	Karagiannis	8	Brozos	GRE		Aristotle U Thessaloniki			Original	Field	Risk factors		Dairy	
416	Ultrasonographic findings in the ovine udder during involution	2014	J Dairy Res	Petridis	7	Fthenakis	GRE		U Thessaly			Original	Experimental	Pathogenesis		Dairy	
417	Identifying the major bacteria causing intramammary infections in individual milk samples of sheep and goats using traditional bacteria culturing and real-time polymerase chain reaction	2014	J Dairy Sci	Rovai	7	Leitner	ISR	ESP	Kimron Veterinary Institute	U Barcelona		Original	Laboratory	Aetiology		Dairy	
418	Comparison of bacteriological culture and PCR for detection of bacteria in ovine milk -Sheep are not small cows	2014	J Dairy Sci	Zadoks	7	Ballingall	GBR		Moredun Research Institute	U Glasgow		Original	Laboratory	Aetiology		Meat	
419	Staphylococcus aureus from 152 cases of bovine, ovine and caprine mastitis investigated by Multiple-locus variable number of tandem repeat analysis (MLVA)	2014	Vet Res	Bergonier	10	Vergnaud	FRA		U Toulouse	INRA	U Paris	Original	Laboratory	Aetiology		Dairy	
420	Sequence diversity, cytotoxicity and antigenic similarities of the leukotoxin of isolates of Mannheimia species from mastitis in domestic sheep	2014	Vet Microbiol	Omaleki	6	Markham	AUS		U Melbourne			Original	Laboratory	Aetiology		Meat	
421	Yeasts in the raw ewe's milk	2014	Acta Scient Vet	Spanaberg	8	Santurio	BRA		U Rio Grande do Sul	U Santa Maria		Original	Field	Aetiology		Dairy	
422	Effects of lactation stage, lactation order and udder types on udder traits and composition of milk in Tuj ewes	2015	Ank Univers Vet Fakult Derg	Sari	3	Onk	TUR		U Kafkas			Original	Field	Risk factors		Dairy	
423	Sheep milk: physical-chemical characteristics and microbiological quality	2015	Arq Latinoameric Nutr	Merlin	7	de Santana	BRA		U North Parana			Original	Laboratory	Effects		Dairy	
424	Divergence of a strain of Pseudomonas aeruginosa during an outbreak of ovine mastitis	2015	Vet Microbiol	Wright	11	Winstanley	GBR		U Liverpool			Original	Laboratory	Aetiology		Meat	
425	Effects of intramammary antibiotic therapy during the dry period on the performance of Lacaune dairy sheep under intensive management	2015	J Dairy Res	Hernandez	7	Astiz	ESP		INIA	Granja Cerromonte		Original	Field	Control		Dairy	
426	Interactions between parasitic infections and reproductive efficiency in sheep	2015	Vet Parasitol	Fthenakis	4	Papadopoulos	GRE		U Thessaly	Aristotle U Thessaloniki		Review					
427	Effects of intramammary infection on whey proteinograms of sheep during lactation	2015	Pesq Vet Brazil	Lemos	7	de Mendonca	BRA		U Pernambuco			Original	Field	Effects		Dairy	
428	Host-specificity of Staphylococcus aureus causing intramammary infections in dairy animals assessed by genotyping and virulence genes	2015	Vet Microbiol	Bar-Gal	6	Leitner	ISR		Kimron Veterinary Institute	Hebrew U Jerusalem		Original	Laboratory	Aetiology		Dairy	
429	Prevalence, genetic diversity, and antimicrobial susceptibility profiles of Staphylococcus aureus isolated from bulk tank milk from Greek traditional ovine farms	2015	Small Rumin Res	Zdragas	10	Lagka	GRE		Agricultural Organisation Dimitra Greece	TEI Thessaloniki		Original	Laboratory	Aetiology		Dairy	
430	Effect of subclinical intramammary infection on milk quality in dairy sheep: I. Fresh-soft cheese produced from milk of uninfected and infected glands and from their blends	2015	Small Rumin Res	Rovai	5	Leitner	ISR		Kimron Veterinary Institute	U Barcelona		Original	Field	Effects		Dairy	
431	Relationship of somatic cell count and composition and coagulation properties of ewe's milk	2015	Mljekarstvo	Villalobos	7	da Cunha	ESP		U Cordoba			Original	Field	Effects		Dairy	
432	Clonal profile, virulence and resistance of Staphylococcus aureus isolated from sheep milk	2015	Braz J Microbiol	Martins	9	da Cunha	BRA		U Paulista			Original	Laboratory	Aetiology		Dairy	
433	Effect of subclinical intramammary infection on milk quality in dairy sheep: II. Matured-pressed cheese (Manchego) produced from milk of uninfected and infected glands and from their blends	2015	Small Rumin Res	Rovai	10	Leitner	ISR	ESP	Kimron Veterinary Institute	U Barcelona		Original	Field	Effects		Dairy	
434	Genetic selection for reduced somatic cell counts in sheep milk: a review	2015	Small Rumin Res	Riggio	2	Portolano	NED	ITL	U Wageningen	U Palermo		Review					
435	Complete genome sequence of Corynebacterium camporealensis DSM 44610, isolated from the milk of a Manchega sheep with subclinical mastitis	2015	Microbiol Resourc Announc	Ruckert	4	Tauch	GER	USA	U Bielefeld	Massachusetts Institute of Technology		Original	Laboratory	Aetiolog		Dairy	
436	Effect of automatic cluster removers on milking efficiency and teat condition of Manchega ewes	2015	J Dairy Sci	Bueso-Rodenas	5	Diaz	ESP		U Miguel Hernandez			Original	Laboratory	Risk factors		Dairy	
437	Neutrophil extracellular traps in sheep mastitis	2015	Vet Res	Pisanu	9	Addis	ITL		Ponto Conte Research			Original	Laboratory	Pathogenesis		Dairy	
438	Prevalence and antimicrobial resistance of Staphylococcus aureus isolated from raw milk and dairy products	2015	Food Control	Jamali	5	Dadrasnia	MAL		U Malaya			Original	Laboratory	Aetiology	Treatment	Dairy	
439	Is udder ultrasonography a diagnostic tool for subclinical mastitis in sheep?	2015	Small Rumin Res	Hussein	3	Malek	EGY		U Assiut			Original	Field	Diagnosis		Dairy	
440	Composition, proteolysis indices and coagulating properties of ewe milk as affected by bulk tank somatic cell count	2015	J Dairy Res	Marti De Olives	5	Molina	ESP		U Miguel Hernandez	U Valencia		Original	Laboratory	Effects		Dairy	
441	Pregnancy toxaemia as predisposing factor for development of mastitis in sheep during the immediately post-partum period	2015	Small Rumin Res	Barbagianni	10	Fthenakis	GRE		U Thessaly			Original	Experimental	Risk factors		Dairy	
442	Prevalence and etiology of subclinical mastitis in dairy ewes in two seasons in Semnan province	2015	Trop Anim Hlth and Prod	Sani	3	Moezifar	IRN		U Semnan	U Tehran		Original	Field	Aetiology	Epidemiology	Dairy	
443	Bacterial species and their associations with acute and chronic mastitis in suckler ewes	2015	J Dairy Sci	Smith	6	Green	GBR		U Warwick			Original	Field	Aetiology		Meat	
444	The effect of health status of the udder on plasminogen activator activity of milk somatic cells in ovine milk	2015	Small Rumin Res	Charismiadou	5	Politis	GRE		Agricultural U Athens			Original	Field	Pathogenesis		Dairy	
445	A point mutation in suppressor of cytokine signalling 2 (Socs2) increases the susceptibility to inflammation of the mammary gland while associated with higher body weight and size and higher milk production in a sheep model	2015	Plos Gen	Rupp	14	Tosser -Klopp	FRA		INRA	U Toulouse		Original	Field	Risk factors		Dairy	
446	The upper respiratory tract is a natural reservoir of haemolytic Mannheimia species associated with ovine mastitis	2015	Vet Microbiol	Omaleki	5	Barber	AUS		U Melbourne			Original	Field	Aetiology		Meat	
447	Mastitis in sheep -The last 10 years and the future of research	2015	Vet Microbiol	Gelasakis	5	Fthenakis	GRE		U Thessaly			Review					
448	Vaccination schedules in small ruminant farms	2015	Vet Microbiol	Lacasta	6	Fthenakis	ESP	GRE	U Zaragosa	U Thessaly		Review					
449	Use of proteomics in the study of microbial diseases of small ruminants	2015	Vet Microbiol	Katsafadou	4	Fthenakis	GRE		U Thessaly			Review					
450	Genetic resistance to infections in sheep	2015	Vet Microbiol	Bishop	1		GBR		U Edinburgh			Review					
451	Evaluating coagulation properties of milk from dairy sheep with subclinical intramammary infection using near infrared light scatter. A preliminary study	2016	J Food Eng	Abdelgawad	5	Castillo	ESP	EGY	U Barcelona	U Fayoum		Original	Field	Effects		Dairy	
452	Microbiological quality of raw milk in the Czech republic	2016	Czech J Food Sci	Bogdanovicova	6	Karpiskova	CZE		U Brno	Inst Veterinary Research Czechia		Original	Field	Effects		Dairy	
453	Physiological threshold of somatic cell count in milk of Polish health sheep and Polish lowland sheep	2016	Ann Anim Sci	Swiderek	5	Pierzchala	POL		U Warsow	Academy of Sciences Poland		Original	Field	Diagnosis		Meat	
454	New rapid method of DNA isolation from milk somatic cells	2016	Anim Biotechnol	Pokorska	5	Makulska	POL		U Krakow			Original	Laboratory	Pathogenesis		Dairy	Meat
455	Response to dietary-induced energy restriction in dairy sheep divergently selected for resistance or susceptibility to mastitis	2016	J Dairy Sci	Bouvier-Muller	9	Rupp	FRA		INRA	U Toulouse		Original	Field	Risk factors		Dairy	
456	Relationships between somatic cell count and certain udder and teat echo-morphometric measurements in mastitis caused by Staphylococcus aureus in Awassi sheep	2016	Rev Med Vet	Ismail	3	Al-Sheyab	JOD		Jordan U Science Technology			Original	Field	Risk factors		Dairy	
457	Development and test of a portable device to monitor the health status of Sarda breed sheep by the measurement of the milk electrical conductivity	2016	Ital J Anim Sci	Caria	5	Tozzona	ITL		U Sassari			Original	Field	Diagnosis		Dairy	
458	Seasonal dynamics and possible development of total count of microorganisms in sheep's milk	2016	Acta Vet Brno	Klimesova	10	Vondruskova	CZE		Inst Dairy Research Czechia			Original	Field	Effects		Dairy	
459	Relationship of litter size with milk yield, udder morphology and udder health of East Friesian sheep	2016	J Central Europ Agric	Prpic	4	Mioc	CRO		U Zagreb			Original	Field	Risk factors		Dairy	
460	Comparative study on fitness traits and reproductive efficiency in Turcana and Tsigai ewes	2016	Scient Papers D	Gavojdian	4	Kusza	ROM		Inst Res and Dev Sheep & Goats			Original	Field	Risk factors		Dairy	
461	Prevalence and molecular characterization of staphylococci isolated from sheep with subclinical mastitis in West-Azerbaijan province, Iran	2016	Vet Res Forum	Rahman	4	Ardebili	IRN		U Urmia			Original	Field	Aetiology		Dairy	
462	A cross-sectional study of 329 farms in England to identify risk factors for ovine clinical mastitis	2016	Prev Vet Med	Cooper	5	Green	GBR		U Warwick			Original	Field	Risk factors		Meat	
463	Development of a facial expression scale using footrot and mastitis as models of pain in sheep	2016	Appl Anim Behav Sci	McLennan	6	Constantino-Casas	GBR		U Cambridge			Original	Field	Effects		Meat	
464	Clinical evaluation of the use of enrofloxacin against Staphylococcus aureus clinical mastitis in sheep	2016	Small Rumin Res	Attili	6	Cuteri	ITL		U Camerino			Original	Field	Treatment		Dairy	
465	Association study between beta-defensin gene polymorphisms and mastitis resistance in Valle del Belice dairy sheep breed	2016	Small Rumin Res	Tolone	7	Portolano	ITL		U Palermo			Original	Field	Risk factors		Dairy	
466	Staphylococcus aureus isolates from goat and sheep milk seem to be closely related and differ from isolates detected from bovine milk	2016	Front Microbiol	Merz	3	Johler	SWI		U Zurich			Original	Laboratory	Aetiology		Dairy	

467	Effect of farm characteristics and practices on hygienic quality of ovine raw milk used for artisan cheese production in central Italy	2016	Anim Sci J	Carloni	7	Tonucci	ITL		U Urbino	Inst Zooprofilattico Umbria e Marche		Original	Field	Effects		Dairy	
468	A longitudinal study of factors associated with acute and chronic mastitis and their impact on lamb growth rate in 10 suckler sheep flocks in Great Britain	2016	Prev Vet Med	Grant	3	Green	GBR		U Warwick			Original	Field	Risk factors	Effects	Meat	
469	Diagnosis of subclinical mastitis in Santa Ines and Morada Nova sheep in southeastern Brazil	2016	Trop Anim Hlth and Prod	Zafalon	4	Fim	BRA		U Sao Paolo	Agricultural Research Organisation Brasil		Original	Field	Diagnosis		Dairy	
470	Somatic cell counts, chemical composition and coagulation properties of goat and sheep bulk tank milk	2016	Int Dairy J	Leitner	5	Merin	ISR		Kimron Veterinary Institute	Afimilk Israel		Original	Field	Effects		Dairy	
471	Assessment of genetic variation for pathogen-specific mastitis resistance in Valle del Belice dairy sheep	2016	BMC Vet Res	Tolone	6	Portolano	ITL		U Palermo			Original	Field	Risk factors		Dairy	
472	Characterization of Staphylococcus aureus isolates from raw milk sources in Victoria, Australia	2016	BMC Microbiol	MCMillan	5	Fox	AUS		CSIRO			Original	Field	Effects		Dairy	
473	Evaluation of milk cathelicidin for detection of dairy sheep mastitis	2016	J Dairy Sci	Addis	10	Uzzau	ITL		Ponto Conte Research	U Sassari		Original	Field	Diagnosis		Dairy	
474	Survey on small ruminant bacterial mastitis in Italy, 2013-2014	2016	Small Rumin Res	Dore	13	Cannas	ITL		Inst Zooprofilatico Sardegna			Original	Field	Aetiology	Epidemiology	Dairy	
475	Molecular epidemiology of an outbreak of clinical mastitis in sheep caused by Mannheimia haemolytica	2016	Vet Microbiol	Omaleki	5	Barber	AUS		U Melbourne	Ministry of Agriculture Australia		Original	Laboratory	Aetiology		Meat	
476	Use of nanoparticulated antimicrobials to treat subclinical mastitis of ewes during the dry period	2016	Pesq Vet Brazil	Santana	8	Mosqueira	BRA		Agricultural Research Organisation Brasil	U Ouro Preto		Original	Field	Control		Dairy	
477	Predisposing factors and control of bacterial mastitis in dairy ewes	2016	J Hell Vet Med Soc	Bramis	5	Arsenos	GRE		Aristotle U Thessaloniki			Review					
478	Influence of lactation stages and rain periods on subclinical mastitis in meat producing ewes	2016	Cienc Rural	Zafalon	6	Santana	BRA		Agricultural Research Organisation Brasil			Original	Field	Risk factors		Meat	
479	Characterization of resistance to tetracyclines and aminoglycosides of sheep mastitis pathogens: study of the effect of gene content on resistance	2016	J Appl Microbiol	Lollai	4	Piras	ITL		Inst Zooprofilatico Sardegna	Mastitis Reference Centre Italy		Original	Laboratory	Treatment		Dairy	
480	Effect of management factors on reproductive and milk production performance of a dairy sheep breed adapted to low-input management systems	2017	J Hell Vet Med Soc	Tzanidakis	6	Kiossis	GRE		Aristotle U Thessaloniki	Agricultural Organisation Dimitra Greece		Original	Field	Risk factors		Dairy	
481	Correlation between total bacterial and somatic cell counts in bulk tank ewes' milk	2017	J Food Nutr Res	Klimesova	9	Vondruskova	SLK		Inst Dairy Research Czechia			Original	Field	Diagnosis		Dairy	
482	Comparison of biofilm formation by Staphylococcus aureus and Staphylococcus epidermidis strains isolated from sheep milk using three diagnostic methods	2017	Pol J Vet Sci	Vasil	5	Zigo	SLK		U Kosice			Original	Laboratory	Aetiology		Dairy	
483	Relationship of mammary gland health status and other noninfectious factors with electrical conductivity of milk in Manchega ewes	2017	J Dairy Sci	Romero	5	Diaz	ESP		U Miguel Hernandez			Original	Field	Diagnosis		Dairy	
484	Characteristics of resistance and virulence factors in different species of coagulase-negative staphylococci isolated from milk of healthy sheep and animals with subclinical mastitis	2017	J Dairy Sci	Martins	8	Cunha	BRA		U Paulista			Original	Laboratory	Aetiology		Dairy	
485	Occurrence of mastitis and associated pathogens with antibiogram in animal population of Peshawar, Pakistan	2017	Thai J Vet Med	Rafiullah	8	Khan	PAK		Veterinary Research Inst Pakistan			Original	Field	Aetiology	Treatment	Dairy	
486	Serum concentrations of acute phase proteins in goats and ewes with naturally acquired Staphylococcus aureus mastitis	2017	Arq Brasil Med Vet Zoot	Simplicio	6	Fagliari	BRA		U Paulista			Original	Field	Pathogenesis		Dairy	
487	Bacteriological quality and occurrence of some microbial pathogens in goat's and ewe's milk in Egypt	2017	Int Food Res J	Ombarak	2	Elbagory	EGY		U Sadat City			Original	Field	Aetiology		Dairy	
488	Susceptibility and PK/PD relationships of Staphylococcus aureus strains from ovine and caprine with clinical mastitis against five veterinary fluoroquinolones	2017	Vet Rec	Serrano-Rodriguez	6	Fernandez-Varon	ESP		U Cordoba	U Murcia		Original	Laboratory	Treatment		Dairy	
489	B-Mode ultrasonography of mammary glands in dairy ewes during the lactation period	2017	Revist Cientif	Makovicky	3	Margetin	SLK		U J Selye	Agricultural U Slovakia	Inst Research Animal Production Slovakia	Original	Field	Diagnosis		Dairy	
490	Antimicrobial susceptibility and genotyping of Staphylococcus aureus isolates collected between 1986 and 2015 from ovine mastitis	2017	Vet Microbiol	Azara	4	Tola	ITL		Inst Zooprofilatico sardegna			Original	Laboratory	Aetiology	Treatment	Dairy	
491	Ultrasonographic examination of the udder in sheep	2017	Small Rumin Res	Barbagianni	5	Petridis	GRE		U Thessaly			Review					
492	Cathelicidin production and release by mammary epithelial cells during infectious mastitis	2017	Vet Immunol Immunopathol	Cubeddu	11	Addis	ITL		Ponto Conte Research	U Sassari	U Milan	Original	Field	Pathogenesis		Dairy	
493	Effects of intramammary infusion of sage (Salvia officinalis) essential oil on milk somatic cell count, milk composition parameters and selected hematology and serum biochemical parameters in Awassi sheep with subclinical mastitis	2017	Vet World	Alekish	5	Queiroga	JOD		Jordan U Science Technology			Original	Field	Control		Dairy	
494	Prevalence and aetiology of sheep mastitis in Alentejo region of Portugal	2017	Small Rumin Res	Queiroga	1		POR		U Evora			Original	Field	Aetiology	Epidemiology	Dairy	
495	Biofilm formation and virulence factor analysis of Staphylococcus aureus isolates collected from ovine mastitis	2017	J Appl Microbiol	Azara	4	Tola	ITL		Inst Zooprofilatico sardegna			Original	Laboratory	Pathogenesis		Dairy	
496	The genomic architecture of mastitis resistance in dairy sheep	2017	BMC Genomics	Banos	10	Psifidi	GBR	GRE	U Edinburgh	Aristotle U Thessaloniki	U London	Original	Field	Risk factors		Dairy	
497	Somatic cell counts in raw ewes' milk in dairy practice: frequency of distribution and possible effect on milk yield and composition	2017	Mljekarstvo	Tancin	6	oravcova	SLK		Agricultural U Slovakia	Inst Research Animal Production Slovakia		Original	Field	Diagnosis	Effects	Dairy	
498	Prevalence and characterization of methicillin-resistant Staphylococcus aureus carrying mecA or mecC and methicillin-susceptible Staphylococcus aureus in dairy sheep farms in central Italy	2017	J Dairy Sci	Giancinti	13	Battisti	ITL		Inst Zooprofilatico Lazio e Toscana			Original	Laboratory	Treatment		Dairy	
499	Gastrointestinal trichostrongylosis can predispose ewes to clinical mastitis after experimental mammary infection	2017	Vet Parasitol	Mavrogianni	8	Fthenakis	GRE		U Thessaly			Original	Experimental	Risk factors		Dairy	
500	Intramammary infections and somatic cell counts in meat and pelt producing ewes with clinically healthy udders	2017	Small Rumin Res	Persson	5	Waller	SWE		National Veterinary Institute Sweden	U Uppsala	Vaxa Sweden	Original	Field	Effects		Meat	Wool
501	Microbial agents in macroscopically healthy mammary gland tissues of small ruminants	2017	Peer J	Spurria	15	Cappuchio	ITL		U Turin			Original	Field	Aetiology		Dairy	
502	Intramammary infusion of a live culture of Lactococcus lactis in ewes to treat staphylococcal mastitis	2017	J Med Microbiol	Mignacca	14	Marianelli	ITL		Inst Zooprofilatico Sicilia	Institute of Health Italy		Original	Field	Treatment		Dairy	
503	Differential microbiological quality of sheep milk from bulk tank	2018	Rev Sci	Jimenez-Sobrino	5	Arias-Sanchez	ESP		INIA			Original	Field	Effects		Dairy	
504	The relationship between somatic cells and milk traits, and their variation in dairy sheep breeds in Slovakia	2018	J Anim Feed Sci	Oravcova	3	Tancin	SLK		Agricultural U Slovakia	Inst Research Animal Production Slovakia		Original	Field	Risk factors		Dairy	
505	Somatic cell count in the diagnosis of subclinical mastitis in sheep of different breeds	2018	Semina Cien Agrar	Zafalon	4	Fim	BRA		U Paulista	Agricultural Research Organisation Brasil		Original	Field	Diagnosis		Dairy	
506	Antimicrobial activity of essential oils of lamiaceae aromatic spices towards sheep mastitis-causing Staphylococcus aureus and Staphylococcus epidermidis	2018	J Essential Oil Bearing Plants	Queiroga	5	Martins	POR		U Evora			Original	Field	Control		Dairy	
507	Antibacterial effect of Czech and Manuka honey on selected mastitis pathogens	2018	Acta Vet Brno	Klimesova	3	Nejeschlebova	CZE		Inst Dairy Research Czechia			Original	Field	Treatment		Dairy	
508	The effects on contagious mastitis pathogens in bulk tank on physicochemical properties of Iranian white cheese	2018	Karpatian J Food Sci Technol	Gheisari	5	Roozbahani	IRN		U Shiraz			Original	Field	Effects		Dairy	
509	Bacteriological cure rate and changes in milk composition in mastitis vaccinated ewes affected with subclinical mastitis	2018	Vet World	Alekish	6	Olymat	JOD		Jordan U Science Technology			Original	Field	Control		Dairy	
510	Risk factors of teat-end hyperkeratosis and its association with udder health in dairy ewes	2018	J Dairy Res	Vouraki	4	Arsenos	GRE		Aristotle U Thessaloniki			Original	Field	Risk factors		Dairy	
511	Use of contrast-enhanced ultrasonographic examination to evaluate health status of mammary glands of ewes at the end of a lactation period	2018	J Dairy Res	Mantziaras	8	Barbagianni	GRE		U Thessaly	Air Force Greece		Original	Field	Diagnosis		Dairy	
512	First description in Greece of mphC-positive staphylococci causing subclinical mastitis in ewes	2018	Microbial Drug Res	Papagiannitsis	8	Petinaki	GRE	CZE	U Thessaly	U Prague		Original	Laboratory	Treatment		Dairy	
513	Somatic cell count-based selection reduces susceptibility to energy shortage during early lactation in a sheep model	2018	J Dairy Sci	Bouvier-Muller	7	Rupp	FRA		U Toulouse	INRA		Original	Field	Risk factors		Dairy	
514	Isolation and identification of bacteria causing mastitis in small ruminants and their susceptibility to antibiotics, honey, essential oils, and plant extracts	2018	Vet World	Abdalhamed	3	Abou Zeina	EGY		Nation Research Institute Egypt			Original	Laboratory	Treatment		Dairy	
515	Relationship between virulence factor genes in coagulase-negative Staphylococcus spp. and failure of antimicrobial treatment of subclinical mastitis in sheep	2018	Pesq Vet Brazil	Zafalon	8	Pilon	BRA		U Paulista	Agricultural Research Organisation Brasil		Original	Laboratory	Treatment		Dairy	
516	Phenotypic and genetic relationships between indicators of the mammary gland health status and milk composition, coagulation, and curd firming in dairy sheep	2018	J Dairy Sci	Pazzola	6	Vacca	ITL		U Sassari			Original	Field	Risk factors		Dairy	
517	Determination of milk production losses and variations of fat and protein percentages according to different levels of somatic cell count in Valle del Belice dairy sheep	2018	Small Rumin Res	Sutera	6	Tolone	ITL		U Palermo			Original	Field	Effects		Dairy	
518	Antibiotic-resistance, enterotoxin gene profiles and farm-level prevalence of Staphylococcus aureus in cow, sheep and goat bulk tank milk in Jordan	2018	Int Dairy J	Obaidat	4	Al-Hakimi	JOD		Jordan U Science Technology			Original	Laboratory	Treatment		Dairy	
519	The mammary microenvironment in mastitis in humans, dairy ruminants, rabbits and rodents: a one health focus	2018	J Mammary Gland Biol Neopl	Hueghs	2	Watson	GBR		U Cambridge			Review					
520	Association between polymorphism of ABCG2 gene and somatic cell count in Czech dairy sheep breeds	2018	Med Wet	Hofmannova	6	Svitakova	CZE		Inst Animal Science Czechia			Original	Field	Risk factors		Dairy	
521	Extensive countrywide field investigation of subclinical mastitis in sheep in Greece	2018	J Dairy Sci	Vasileiou	13	Fthenakis	GRE		U Thessaly			Original	Field	Aetiology	Epidemiology	Dairy	
522	Association of subclinical mastitis prevalence with sheep breeds in Greece	2018	J Dairy Res	Vasileiou	8	Fthenakis	GRE		U Thessaly			Original	Field	Risk factors		Dairy	
523	Reference values for serum amyloid A, haptoglobin, lysozyme, zinc and iron in healthy lactating Lacaune sheep	2018	Acta Vet Scand	Miglio	6	Felici	ITL		U Perugia	Inst Zooprofilattico Umbria e Marche		Original	Laboratory	Pathogenesis		Dairy	
524	Diagnostic evaluation of milk lactate dehydrogenase and alkaline phosphatase activities by receiver operating characteristic analysis curve in early lactation of ewes with subclinical mastitis	2018	Vet Res Forum	Sani	4	Kafshdouzan	IRN		U Semnan			Original	Laboratory	Diagnosis		Dairy	
525	Factors affecting ewe somatic cell count and its relationship with lamb weaning weight in extensively managed flocks	2018	Translational Anim Sci	Murthy	3	Taylor	USA		U Montana State	Ministry of Agriculture USA		Original	Field	Risk factors	Effects	Meat	
526	Local and systemic humoral response to ovine mastitis caused by Staphylococcus epidermidis	2018	Sage Open Med	Queiroga	1		POR		U Evora			Original	Laboratory	Pathogenesis		Dairy	
527	Detection of quantitative trait loci and putative causal variants affecting somatic cell score in dairy sheep by using a 50K SNP chip and whole-genome sequencing	2018	J Dairy Sci	Gutierrez-Gil	4	Arranz	ESP		U Leon			Original	Laboratory	Risk factors		Dairy	
528	Udder types and associated traits affect milk composition and subclinical mastitis in Karayaka sheep	2018	Ind J Anim Sci	Akdag	6	Demir	TUR		U Ondokuz Mayis	U Cerrahpasa		Original	Field	Risk factors		Dairy	
529	Antimicrobial resistance in mastitis, respiratory and enteric bacteria isolated from ruminant animals from the Atlantic Provinces of Canada from 1994-2013	2018	Can Vet J	Awosile	4	McClure	CDN		U Prince Edward Island			Original	Laboratory	Treatment		Dairy	
530	In vitro antibacterial effects of zinc oxide nanoparticles on multiple drug-resistant strains of Staphylococcus aureus and Escherichia coli: an alternative approach for antibacterial therapy of mastitis in sheep	2018	Vet World	Alekish	4	Nawasrah	JOD		Jordan U Science Technology			Original	Field	Treatment		Dairy	
531	Slime-producing staphylococci as causal agents of subclinical mastitis in sheep	2018	Vet Microbiol	Vasileiou	9	Fthenakis	GRE		U Thessaly			Original	Field	Aetiology	Epidemiology	Dairy	
532	Lactoferrin and IgG levels in ovine milk throughout lactation: Correlation with milk quality parameters	2018	Small Rumin Res	Navaro	7	Sanchez	ESP	VEN	U Zaragosa	U Los Andes		Original	Laboratory	Diagnosis		Dairy	
533	Sheep mastitis Staphylococcus epidermidis biofilm effects on cell adhesion and inflammatory changes	2018	Small Rumin Res	Queiroga	3	Laranjo	POR		U Evora			Original	Laboratory	Pathogenesis		Dairy	

534	Milk cellularity and intramammary infections in primiparous and multiparous Lacaune ewes during early lactation	2018	Small Rumin Res	Takano	12	Blagitz	BRA	U Sao Paolo		Original	Field	Epidemiology	Diagnosis	Dairy
535	New mastitis phenotypes suitable for genomic selection in meat sheep and their genetic relationships with udder conformation and lamb live weights	2018	Animal	McLaren	6	Conington	GBR	Scotland's Rural College		Original	Field	Risk factors		Meat
536	Mammary gland health of Santa Ines ewes at the drying and puerperium and evaluation of a dry-off terapy with gentamicin	2018	Pesq Vet Brazil	Pereira	8	Muller	BRA	U Londrina		Original	Field	Control		Dairy
537	Use of geographical information system and ecological niche modelling for predicting potential space distribution of subclinical mastitis in ewes	2019	Vet Microbiol	Giannokopoulos	10	Fthenakis	GRE	U Thessaly		Original	Field	Epidemiology	Risk factors	Dairy
538	Field evidence for association between increased gastrointestinal nematode burden and subclinical mastitis in dairy sheep	2019	Vet Parasitol	Kordalis	7	Fthenakis	GRE	U Thessaly		Original	Field	Risk factors		Dairy
539	Associations between lamb growth to weaning and dam udder and teat scores	2019	NZ Vet J	Griffiths	5	Kenyon	NZL	U Massey		Original	Field	Effects		Meat
540	Investigating associations between lamb survival to weaning and dam udder and teat scores	2019	NZ Vet J	Griffiths	5	Kenyon	NZL	U Massey		Original	Field	Effects		Meat
541	Milk electrical conductivity in Manchega ewes: variation throughout milking and relation with mammary gland health status	2019	Czech Anim Sci	Roca	5	Diaz	ESP	U Miguel Hernandez		Original	Field	Diagnosis		Dairy
542	Efficacy of a terpinen-4-ol based dipping for post-milking teat disinfection in the prevention of mastitis in dairy sheep	2019	J Essential Oil Res	Dore	7	Cannas	ITL	Mastitis Reference Centre Italy		Original	Field	Control		Dairy
543	Study of potential environmental factors predisposing ewes to subclinical mastitis in Greece	2019	Comp Immunol Microbiol Inf Dis	Vasileiou	10	Fthenakis	GRE	U Thessaly		Original	Field	Risk factors		Dairy
544	An approach for the estimation of somatic cells' effect in Sarda sheep milk based on the analysis of milk traits and coagulation properties	2019	Small Rumin Res	Paschino	4	Pazzola	ITL	U Sassari		Original	Field	Effects		Dairy
545	Antimicrobial susceptibility pattern to disinfectants in Pseudomonas aeruginosa strains isolated from dairy sheep breeds in Sardinia	2019	Large Anim Rev	Scano	12	Liciardi	ITL	U Cagliari	Inst Zooprofilatico Sardegna	Original	Laboratory	Control		Dairy
546	A validation study of loci associated with mastitis resistance in two French dairy sheep breeds	2019	Gen Sel Evol	Oget	9	Rupp	FRA	U Toulouse		Original	Field	Risk factors		Dairy
547	Antimicrobial agent susceptibility and typing of staphylococcal isolates from subclinical mastitis in ewes	2019	Microbial Drug Res	Vasileiou	8	Fthenakis	GRE	U Thessaly		Original	Laboratory	Treatment		Dairy
548	Biofilm production, quorum sensing system and analysis of virulence factors of Staphylococcus epidermidis collected from sheep milk samples	2019	Small Rumin Res	Abbondio	5	Tola	ITL	Inst zooprofilatico Sardegna		Original	Laboratory	Pathogenesis		Dairy
549	Differences between Staphylococcus aureus lineages isolated from ovine and caprine mastitis but not between isolates from clinical or subclinical mastitis	2019	J Dairy Sci	Hoekstra	6	Koop	NED	U Utrecht		Original	Laboratory	Aetiology		Dairy
550	Proteomic analyses of mammary glands provide insight into the immunity and metabolism pathways associated with clinical mastitis in meat sheep	2019	Animals	Gao	6	Ma	CHN	U Gansu	Labor Sheep Breedind Gansu China	Original	Laboratory	Pathogenesis		Meat
551	Antimicrobial resistance and distribution of Staphylococcus spp. pulsotypes isolated from goat and sheep bulk tank milk in Southern Spain	2019	Foodborne Pathog Dis	Barrero-Dominguez	7	Astorga	ESP	U Cordoba		Original	Laboratory	Treatment		Dairy
552	Comparative evaluation of metallic skin staples or polypropylene sutures for primary closure of teat wounds in sheep	2019	NZ Vet J	Tsioli	9	Galatos	GRE	U Thessaly		Original	Experimental	Treatment		Dairy
553	Genetic and genomic studies in ovine mastitis	2019	Small Rumin Res	Oget	3	Rupp	FRA	U Toulouse	INRA	Review				
554	Somatic cell counts in sheep milk	2019	Small Rumin Res	Albenzio	6	Santillo	ITL	U Foggia		Review				
555	Milking routines and cluster detachment levels in small ruminants	2019	Animal	Dzidic	5	Marnet	CRO	U Zagreb	Agrocampus Ouest	Review				
556	Digital gene expression analyses of mammary glands from meat ewes naturally infected with clinical mastitis	2019	R Soc Open Sci	Li	4	Ma	CHN	U Gansu		Original	Laboratory	Risk factors		Meat
557	The relationship between lactoferrin gene polymorphism and subclinical mastitis in Awassi ewes	2019	J Anim Plant Sci	Alekish	4	Alshehabat	JOD	Jordan U Science Technology		Original	Field	Risk factors		Dairy
558	Heritability of phenotypic udder traits to improve resilience to mastitis in Texel ewes	2019	Animal	Crump	5	Green	GBR	U Warwick		Original	Field	Risk factors		Meat
559	Role of staphylococci in mastitis in sheep	2019	J Dairy Res	Vasileiou	8	Fthenakis	GRE	U Thessaly		Review				
560	Proteomics data of ovine mastitis associated with Mannheimia haemolytica	2019	D Brief	Katsafadou	9	Fthenakis	GRE	U Thessaly	Academy Athens Greece	Original	Field	Pathogenesis		Dairy
561	Differential quantitative proteomics study of experimental Mannheimia haemolytica mastitis in sheep	2019	J Proteom	Katsafadou	9	Fthenakis	GRE	U Thessaly	Academy Athens Greece	Original	Experimental	Pathogenesis		Dairy
562	Predisposing factors for bacterial mastitis in ewes	2019	Reprod Dom Anim	Vasileiou	4	Fthenakis	GRE	U Thessaly		Review				
563	Use of proteomics in the study of mastitis in ewes	2019	Pathogens	Katsafadou	3	Fthenakis	GRE	U Thessaly		Review				
564	Anti-staphylococcal biofilm antibodies in ewes and association with subclinical mastitis	2019	Small Rumin Res	Vasileiou	6	Fthenakis	GRE	U Thessaly		Original	Laboratory	Pathogenesis		Dairy
565	Bulk tank somatic cell count and total bacterial count are affected by target practices and milking machine features in dairy sheep flocks in Castilla y Leon region, Spain	2019	Small Rumin Res	Gonzalo	4	de la Fuente	ESP	U Leon		Original	Field	Risk factors		Dairy
566	Alternative methods improve the accuracy of genomic prediction using information from a causal point mutation in a dairy sheep model	2019	BMC Genomics	Oget	5	Rupp	FRA	U Toulouse	INRA	Original	Field	Risk factors		Dairy
567	Evaluation of efficacy of a biofilm-embedded bacteria-based vaccine against staphylococcal mastitis in sheep-A randomized, placebo-controlled field study	2019	J Dairy Sci	Vasileiou	15	Fthenakis	GRE	U Thessaly		Original	Field	Control		Dairy
568	Metataxonomic and immunological analysis of milk from ewes with or without a history of mastitis	2019	J Dairy Sci	Castro	8	Rodriguez	ESP	U Madrid		Original	Laboratory	Risk factors	Pathogenesis	Dairy
569	Gelsolin expression in sheep milk somatic cells during lactation	2019	Animal	Napolitano	8	Marchitelli	ITL	National Research Council Italy		Original	Laboratory	Risk factors		Dairy
570	Mammary defences and immunity against mastitis in sheep	2019	Animals	Katsafadou	7	Fragkou	GRE	U Thessaly		Review				
572	Clinical and subclinical intrammamay infection caused by coagulase negative staphylococci negatively affect milk yield and its quality in dairy sheep	2019	Small Rumin Res	Leitner	3	Merin	ISR	Kimron Veterinary Institute	Agri Research Organisation Israel	Review				
573	Interactions between parasitism and milk production - mastitis in sheep	2019	Small Rumin Res	Vasileiou	7	Papadopoulos	GRE	U Thessaly	Aristotle U Thessaloniki	Review				
574	Comparison of PCR-RFLP, API (R) 20 Strep and MALDI-TOF MS for identification of Streptococcus spp. collected from sheep and goat milk samples	2019	Small Rumin Res	Rosa	4	Tola	ITL	Inst zooprofilatico Sardegna		Original	Laboratory	Aetiology		Dairy
575	Udder surgery in ewes	2019	Small Rumin Res	Tsioli	2	Fthenakis	GRE	U Thessaly		Review				
576	Mammary involution and relevant udder health management in sheep	2019	Small Rumin Res	Petridis	2	Fthenakis	GRE	U Thessaly		Review				
577	Mastitis in ewes: somatic cell counts, pathogens and antibiotic resistance	2019	J Microbiol Biotechnol Food Sci	Tvarozkova	5	Macuhova	SLV	Agricultural U Slovakia	Inst Research Animal Production Slovakia	Review				
578	Subclinical mastitis in Lacaune sheep: causative agents, impacts on milk production, milk quality, oxidative profiles and treatment efficacy of ceftiofur	2019	Microb Pathogen	Alba	10	da Silva	BRA	U Santa Catarina		Original	Field	Aetiology	Treatment	Dairy
579	Experimental study for evaluation of the efficacy of a biofilm-embedded bacteria-based vaccine against Staphylococcus chromogenes-associated mastitis in sheep	2019	Vet Microbiol	Vasileiou	13	Fthenakis	GRE	U Thessaly		Original	Experimental	Control		Dairy
580	Detection of cathelicidin-1 in the milk as an early indicator of mastitis in ewes	2019	Pathogens	Katsafadou	12	Fthenakis	GRE	U Thessaly	Academy Athens Greece	Original	Experimental	Diagnosis		Dairy
581	Subclinical mastitis in sheep: etiology and association with milk somatic cell count and ewe productivity in three research flocks in the Western United States	2019	Translational Anim Sci	Knuth	10	Murphy	USA	U Montana State	Ministry of Agriculture USA	Original	Field	Aetiology	Effects	Dairy

AFSSA: French Agency of Safety of Foods, AGRIS Sardegna: Agricultural Research Agency of Sardinia Italy, ANSES: National Agency for Food, Environmental and Occupational Health & Safety France, CSIRO: Commonwealth Scientific and Industrial Research Organisation Australia, INIA: National Institute of Agricultural Investigations and Technology Spain, INRA: National Institute of Agronomic Research France, Inst: Institute, NEIKER: Basque Institute of Agricultural Investigations Spain, SIA-DGA Zaragoza: Agricutlural Research Service of Aragon Spain, TEI: Technological Educational Institution, U: University

Table S2. Alphabetical list of countries from which papers on ovine mastitis have been published during the period 1970 - 2019.

Country	No. papers published	Country	No. papers published
Algeria	1	Italy	78
Argentina	1	Jordan	12
Australia	24	Malaysia	1
Austria	17	The Netherlands	9
Bangladesh	1	Norway	8
Brazil	40	New Zealand	5
Bulgaria	1	Pakistan	1
Canada	8	Poland	7
China	2	Portugal	9
Croatia	3	Republic of South Africa	1
Cyprus	1	Romania	1
Czech Republic	6	Slovakia	9
Denmark	3	Slovenia	5
Egypt	9	South Korea	1
Finland	1	Spain	87
France	31	Sweden	8
Germany	11	Switzerland	6
Greece	87	Turkey	17
India	4	United Kingdom	50
Iran	8	United States of America	26
Iraq	3	Venezuela	1
Israel	23		

Table S3. Alphabetical list of scientific establishments from which at least 5 papers on ovine mastitis have been published during the period 1970 - 2019.

Establishment	Country	No. of papers published
Agricultural Research Organisation of Brazil	Brazil	6
Agricultural Research Organisation of Israel	Israel	9
Agricultural Research Service of Aragon	Spain	10
Agricultural University of Uppsala	Sweden	8
Agrocampus Ouest	France	6
Aristotle University of Thessaloniki	Greece	21
Autonomous University of Barcelona	Spain	5
Commonwealth Scientific and Industrial Research Organisation	Australia	16
Complutense University of Madrid	Spain	18
Federal University Paulista	Brazil	5
Federal University of Pernambuco	Brazil	6
Jordan University of Science and Technology	Jordan	12
Kimron Veterinary Institute	Israel	17
National Institute of Agronomic Research	France	21
Polytechnic University of Valencia	Spain	11
Technological Educational Institution of Epirus	Greece	6
University Miguel Hernandez de Elche	Spain	6
University Mustafa Kemal	Turkey	5
University of Edinburgh	United Kingdom	6
University of Foggia	Italy	13
University of Leon	Spain	25
University of London	United Kingdom	25
University of Melbourne	Australia	7
University of Milan	Italy	6
University of Palermo	Italy	15
University of Sao Paulo	Brazil	10
University of Sassari	Italy	12
University of Thessaly	Greece	64
University of Toulouse	France	13
University of Wageningen	The Netherlands	5
University of Warwick	United Kingdom	6
University of Zaragoza	Spain	8
University of Zurich	Switzerland	5
Veterinary University of Vienna	Austria	18
Zooprophylactic Institute of Sardinia	Italy	14

Table S4. Classification of papers on ovine mastitis published during the period 1970 – 2019, according to paper type, sheep production system, study type and mastitis aspect covered therein.

Original papers n=339	Dairy production n=428	Experimental n=43	Aetiology n=2
			Control n=7
			Diagnosis n=2
			Effects n=5
			Pathogenesis n=22
			Risk factors n=7
			Treatment n=3
		Field n=272	Aetiology n=58
			Control n=19
			D. epidemiology n=28
			Diagnosis n=63
			Effects n=44
			Pathogenesis n=18
			Risk factors n=73
			Treatment n=16
		Laboratory n=113	Aetiology n=51
			Control n=1
			Diagnosis n=9
			Effects n=2
			Pathogenesis n=27
			Risk factors n=6
			Treatment n=27
	Meat production n=113	Experimental n=43	Control n=6
			Diagnosis n=2
			Effects n=1
			Pathogenesis n=16
		Field n=58	Aetiology n=17
			Control n=3
			D. epidemiology n=4
			Diagnosis n=11
			Effects n=14
			Pathogenesis n=4
			Risk factors n=13
		Laboratory n=31	Aetiology n=18
			Diagnosis n=2
			Pathogenesis n=6
			Risk factors n=1
			Treatment n=4
	Wool production n=1	Field n=1	Effects n=1
Review papers n=41			

Table S5. Number of papers on ovine mastitis published during the period 1970 – 2019 originating from the 11 countries with highest number of papers published, classified according to paper type, sheep production system, study type and mastitis aspect covered.

Country	Paper type		Production system referred to			Study type			Mastitis aspect covered							
	Original	Review	Dairy	Meat	Wool	Experim-ental	Field	Labora-tory	Aetiology	Control	D. epide-miology	Diagnosis	Effects	Patho-genesis	Risk factors	Treat-ment
Australia	23	1	2	21	0	14	4	5	5	4	1	2	1	11	0	0
Austria	16	1	9	7	0	4	11	1	5	1	1	4	2	5	0	1
Brazil	39	1	35	4	0	2	24	13	15	3	3	9	1	5	4	6
France	27	4	27	0	0	1	11	15	8	0	2	2	0	7	9	1
Greece	71	16	71	0	0	22	41	8	9	5	8	8	4	21	20	5
Israel	22	1	22	0	0	2	15	5	5	4	1	2	9	2	0	2
Italy	76	2	75	1	0	3	54	19	12	3	3	12	9	12	21	10
Spain	84	3	83	1	0	10	48	26	26	6	15	15	2	11	20	5
Turkey	16	1	16	0	0	0	8	8	8	1	1	4	0	0	2	2
United Kingdom	43	7	5	38	0	3	24	16	14	1	1	5	7	9	8	2
United States of America	24	2	9	15	0	2	16	6	10	2	2	2	7	3	2	0
P	0.0071			<0.0001			<0.0001					<0.0001				

Table S6. Associations between journals and countries of origin of papers on ovine mastitis published during the period 1970 - 2019.

**(a) Country origin of papers published in the most used journals**	
**Journal**	**Two countries with the most papers published therein**
Journal of Dairy Research	Greece n=10, Italy n=4
Journal of Dairy Science	Spain n=26, Italy n=9
Journal of Veterinary Medicine B	Greece / Spain n=2 (each)
Pesquisa Veterinária Brasileira	Brazil n=14
Small Ruminant Research	Greece n=28, Italy n=21
The Veterinary Journal	United Kingdom n=6, Greece n=2
The Veterinary Record	United Kingdom n=6, France / Greece / Spain n=4 (each)
Veterinary Microbiology	Greece n=8, Spain n=6

**(b) Most used journals by authors from the 11 countries with highest number of papers published**	
**Country**	**Two journals with most papers published therein**
Australia	*Research in Veterinary Science* / *Veterinary Microbiology* n=4 (each)
Austria	*Wiener Tierärztliche Monatsschrift* n=4
Brazil	*Pesquisa Veterinária Brasileira* n=14, *Arquivo Brasileiro de Medicina Veterinária e Zootecnia* n=5
France	*Small Ruminant Research* n=5, *Journal of Dairy Science* n=4
Greece	*Small Ruminant Research* n=28, *Journal of Dairy Research* n=10
Israel	*Small Ruminant Research* n=8, *South African Journal of Animal Science* n=3
Italy	*Small Ruminant Research* n=21, *Italian Journal of Animal Science* n=3
Spain	*Journal of Dairy Science* n=26, *Small Ruminant Research* n=11
Turkey	*Kafkas Üniversitesi Veteriner Fakültesi Dergisi* n=4, *Bulletin of the Veterinary Institute in Pulawy* / *Tropical Animal Health and Production* / *Turkish Journal of Veterinary and Animal Sciences* n=2 (each)
United Kingdom	*Journal of Dairy Science* /*The Veterinary Record* / *The Veterinary Journal* n=6 (each)
United States of America	*Journal of Animal Science* n=5, *Small Ruminant Research* n=4

Table S7. Number of papers on ovine mastitis published during the period 1970 – 2019 in the 8 journals with the majority of relevant papers, classified according to paper type, sheep production system, study type and mastitis aspect covered.

Journal	Paper type		Production system referred to			Study type			Mastitis aspect covered							
	Original	Review	Dairy	Meat	Wool	Experim-ental	Field	Labora-tory	Aetiology	Control	D. epide-miology	Diagnosis	Effects	Patho-genesis	Risk factors	Treat-ment
Journal of Dairy Research	20	2	18	2	0	3	13	4	4	2	0	5	3	4	4	0
Journal of Dairy Science	54	0	48	6	0	5	38	11	13	3	0	13	9	3	19	2
Journal of Veterinary Medicine B	11	0	7	4	0	3	4	4	5	0	0	1	0	6	0	0
Pesquisa Veterinária Brasileira	14	0	13	1	0	2	8	4	3	2	0	3	2	2	1	4
Small Ruminant Research	76	14	71	5	1	4	60	12	20	6	12	18	10	9	14	4
The Veterinary Journal	10	1	4	6	0	4	4	2	1	0	2	3	2	2	0	0
The Veterinary Record	13	1	7	6	0	1	11	1	3	1	1	2	1	2	2	2
Veterinary Microbiology	24	5	18	6	0	5	3	16	16	2	2	1	0	5	2	2
P	0.0445		0.0007			<0.0001			0.0001							

Table S8. Literature references in papers on ovine mastitis published during the period 1970 – 2019, according to year of publication, country of origin, establishment of origin, journal in which published and characteristic of paper.

**Year of publication**	**References per paper (x±se)**
1970s	20.0±3.7
1980s	16.4±1.9^{a,b}
1990s	21.0±1.1^{c,d}
2000s	30.8±1.6^{a,c,e}
2010s	41.4±1.6^{b,d,e}
P	<0.0001
**Country of origin**	**References per paper (x±se)**
Australia	27.5±2.5^{a}
Austria	29.5±2.4
Brazil	37.1±3.3
France	50.6±6.3^{a,b,c,d,e}
Greece	43.5±3.9^{f,g}
Israel	26.4±3.0^{e,f}
Italy	35.0±2.0
Spain	32.6±2.6^{b}
Turkey	28.8±2.1
United Kingdom	27.5±3.5^{c,g}
United States of America	28.4±3.1^{d}
P	0.0002
**Establishment of origin**	**References per paper (x±se)**
Aristotle University of Thessaloniki	39.6±7.8
Commonwealth Scientific and Industrial Research Organisation	27.1±3.2
Complutense University of Madrid	25.7±2.3
Jordan University of Science and Technology	25.7±4.3
Kimron Veterianry Institute	28.2±3.7
National Institute of Agronomic Research	50.0±5.7
Polytechnic University of Valencia	30.8±2.9
University of Foggia	39.7±2.9
University of Leon	33.8±4.8
University of London	20.3±3.0
University of Palermo	35.2±6.0
University of Sassari	35.2±5.7
University of Thessaly	47.7±5.0
University of Toulouse	61.6±9.7
Veterinary University of Vienna	29.0±2.3
Zooprophylactic Institute of Sardinia	26.9±2.9
P	<0.0001
**Journal**	**References per paper (x±se)**
Journal of Dairy Research	35.5±6.0
Journal of Dairy Science	35.4±3.6
Journal of Veterinary Medicine B	20.2±2.3
Pesquisa Veterinária Brasileira	44.9±3.7
Small Ruminant Research	36.3±2.6
The Veterinary Journal	19.3±3.4
The Veterinary Record	20.3±3.7^{a}
Veterinary Microbiology	43.6±7.0^{a}
P	0.0034

Table S8 (continued)

**Type of paper**	
Original	29.6±0.7
Review	81.8±7.1
P	<0.0001
**Production system**	
Dairy	31.4±0.8
Meat	22.5±0.8
Wool	21.0
P	<0.0001
**Type of work**	
Experimental	30.7±2.3
Field	28.2±0.8[a]
Laboratory	32.2±1.4[a]
P	0.0310
**Aspect of mastitis covered**	
Aetiology	29.4±1.1
Control	26.5±2.0
D. epidemiology	29.2±2.9
Diagnosis	28.0±1.6
Effects	29.5±1.7
Pathogenesis	32.2±2.3
Risk factors	31.3±1.5
Treatment	28.02±2.0
P	0.48

Table S9. Citations received by papers on ovine mastitis published during the period 1970 – 2019, and impact of the respective journal, according to year of publication, country of origin, establishment of origin, journal in which published and type of paper, production system, type of study and aspect of mastitis covered.

Classification	Scimago category			Citations per paper (x±se)	Citations per paper yearly	Total cites
	Q1	Q2	≥Q3			
**Year of publications**		(n)		(x±se)	(x±se)	(n)
1970s	6	0	2	13.7±5.5	0.3±0.1	109
1980s	9	9	4	19.8±3.0	0.6±0.1[a]	435
1990s	42	45	21	22.1±2.3[a]	0.9±0.1[b,c]	2407
2000s	70	73	25	26.8±2.9[b]	1.8±0.2[b]	4495
2010s	113	103	57	8.4±0.6[a,b]	1.9±0.1[a,c]	2300
P		0.37		<0.0001	0.0001	
**Country of origin**		(n)		(x±se)	(x±se)	(n)
Australia	13	9	2	15.3±1.8[a]	1.0±0.2[a]	368
Austria	4	5	8	12.8±2.5[b]	0.7±0.1[b]	217
Brazil	2	13	25	6.4±1.3[c,d]	1.0±0.2[c]	254
France	23	6	2	38.6±11.1[a,b,c,e,f,g]	3.2±0.7[a,b,c,d,e,f]	1196
Greece	34	50	3	13.1±1.9[d,e]	2.2±0.3	1143
Israel	10	10	3	28.0±6.4	2.5±0.5	643
Italy	34	41	3	14.5±1.8[f]	1.8±0.2	1132
Spain	55	25	7	24.7±3.2	1.7±0.2[d]	2148
Turkey	1	7	9	7.2±1.6[g]	0.8±0.2[e]	123
United Kingdom	26	23	1	19.3±3.4	1.6±0.3[f]	965
United States of America	16	7	3	30.4±5.4	1.8±0.4	791
P		<0.0001		0.0001	0.0004	
**Establishment of origin**		(n)		(x±se)	(x±se)	(n)
Aristotle U. of Thessaloniki	6	13	2	13.5±2.8	1.3±0.3	284
CSIRO	6	9	1	20.0±1.7	1.1±0.3	321
Complutense U. of Madrid	10	7	1	20.9±3.1	1.2±0.3	376
Jordan U. of Science and Technology	3	6	3	8.2±3.5	0.9±0.2	98
Kimron Vet. Inst.	7	8	2	28.8±8.3	2.9±0.6	490
INRA	18	2	1	28.7±6.2	2.8±0.4	603
Polytechnic U. of Valencia	6	2	3	10.5±2.7	0.9±0.3	116
U. of Foggia	4	8	1	30.6±7.1	2.2±0.4	398
U. of Leon	17	7	1	43.4±8.7	2.8±0.6	1085
U. of London	9	17	0	18.2±4.4	0.9±0.2	474
U. of Palermo	8	7	0	7.4±1.6	1.1±0.2	111
U. of Sassari	5	7	0	13.6±2.4	2.6±0.6	163
U. of Thessaly	28	35	1	11.8±1.6	2.5±0.4	756
U. of Toulouse	8	2	0	48.7 ±25.2	4.3±1.4	633
Veterinary U. of Vienna	4	5	9	12.6±2.3	0.7±0.1	226
Zooprophylactic Inst. of Sardinia	4	8	2	12.2±3.7	1.9±0.5	171
P		<0.0001		< 0.0001	0.0435	
**Type of paper**		(n)		(x±se)	(x±se)	(n)
	227	209	103	15.4±0.8	1.4±0.1	8296
Review	13	21	7	35.4±9.9	3.6±0.7	1450
P		0.28		<0.0001	<0.0001	

CSIRO: Commonwealth Scientific and Industrial Research Organisation Australia, INRA: National Institute of Agronomic Research France, U.: University

Table S9 (continued)

Classification	Scimago category			Citations per paper (x±se)	Citations per paper yearly	Total cites (n)
	Q1	Q2	≥Q3			
**Production system**		(n)		(x±se)	(x±se)	(n)
Dairy	171	167	90	15.5±0.9	1.6±0.1	6639
Meat	57	43	13	14.9±1.4	1.0±0.1	1687
Wool	0	1	0	3.0	1.2	3
P		0.085		0.083	0.0031	
**Type of study**		(n)		(x±se)	(x±se)	(n)
Experimental	37	21	7	17.1±2.1	1.4±0.2	1149
Field	116	138	75	15.5±1.1	1.5±0.1	5097
Laboratory	74	50	19	14.3±1.5	1.6±0.2	2050
P		0.0006		0.595	0.610	
**Aspect of mastitis covered**		(n)		(x±se)	(x±se)	(n)
Aetiology	66	55	26	18.7±1.7	1.7±0.2	2736
Control	10	19	7	12.7±2.0	1.0±0.2	458
D. epidemiology	26	38	24	22.8±4.4	2.2±0.6	730
Diagnosis	31	19	16	18.0±2.3	1.2±0.1	1580
Effects	10	15	7	18.8±3.3	1.9±0.3	1239
Pathogenesis	43	35	13	14.1±1.5	1.4±0.2	1283
Risk factors	50	37	13	13.0±1.5	1.6±0.2	1299
Treatment	16	23	11	9.5±1.7	1.3±0.3	477
P		0.067		0.0084	0.407	

Table S10. Citations received by papers on ovine mastitis published during the period 1970 – 2019, according to the journal in which they have been published.

Journal	Citations per paper (x±se)	Yearly citations per paper (x±se)	Total cites (n)
J. Dairy Res.	13.5±2.4	1.6±0.2	296
J. Dairy Sci.	28.9±8.4	2.8±0.8	1556
J. Vet. Med. B	12.2±3.4	0.6±0.2	134
Pesq. Vet. Brasil.	7.2±1.6	0.8±0.2	43
Small Rumin. Res.	22.8±3.3	2.0±0.2	2051
Vet. J.	15.1±3.5	0.9±0.2	404
Vet. Rec.	36.7±8.4	1.5±0.2	212
Vet Microbiol.	18.4±4.0	2.8±0.6	533
P	0.0183	0.0015	

Abbreviations of journals from top to bottom: *Journal of Dairy Research, Journal of Dairy Science, Journal of Veterinary Medicine B, Pesquisa Veterinária Brasileira, Small Ruminant Research, The Veterinary Journal, The Veterinary Record, Veterinary Microbiology*

© 2020 by the authors. Licensee MDPI, Basel, Switzerland. This article is an open access article distributed under the terms and conditions of the Creative Commons Attribution (http://creativecommons.org/licenses/by/4.0/).
